# BRD7 suppresses invasion and metastasis in breast cancer by negatively regulating YB1-induced epithelial-mesenchymal transition

**DOI:** 10.1186/s13046-019-1493-4

**Published:** 2020-02-07

**Authors:** Weihong Niu, Yanwei Luo, Yao Zhou, Mengna Li, Chunchun Wu, Yumei Duan, Heran Wang, Songqing Fan, Zheng Li, Wei Xiong, Xiaoling Li, Guiyuan Li, Caiping Ren, Hui Li, Ming Zhou

**Affiliations:** 1 The Affiliated Tumor Hospital of Xiangya Medical School, Central South University, Changsha, Hunan 410013 People’s Republic of China; 2 Cancer Research Institute, School of Basic Medical Sciences, Central South University, Changsha, Hunan 410078 People’s Republic of China; 3 The Key Laboratory of Carcinogenesis of the Chinese Ministry of Health, The Key Laboratory of Carcinogenesis and Cancer Invasion of the Chinese Ministry of Education, Central South University, Changsha, Hunan 410078 People’s Republic of China; 4 The Second Xiang-Ya Hospital, Central South University, Changsha, Hunan 410011 People’s Republic of China; 5 High Resolution Mass Spectrometry Laboratory of Advanced Research Center, Central South University, Changsha, Hunan 410013 People’s Republic of China

**Keywords:** Breast cancer, BRD7, YB1, EMT, Invasion and metastasis

## Abstract

**Background:**

BRD7 is a tumor suppressor known to inhibit cell proliferation and cell cycle progression and initiate apoptosis in breast cancer. However, the function and underlying molecular events of BRD7 in tumor invasion and metastasis in breast cancer are not fully understood.

**Methods:**

BRD7 expression was assessed in two stable cell lines MDA231 and MCF7 with BRD7 overexpression and one stable cell line MDA231 with BRD7 interference using qRT-PCR and western blotting. CCK8 assay was used to examine the proliferation ability of MDA231 and MCF7 cells. Scratch wound healing assay was used to evaluate cell migration in MDA231 and MCF7 cells. Both Matrigel and three-dimensional invasion assays were performed to investigate the cell invasion ability after BRD7 overexpression or silencing or YB1 restoration in MDA231 and MCF7 cells. The potential interacting proteins of BRD7 were screened using co-immunoprecipitation combined with mass spectrometry and verified by co-immunoprecipitation in HEK293T cells. Additionally, we confirmed the specific binding region between BRD7 and YB1 in HEK293T cells by constructing a series of deletion mutants of BRD7 and YB1 respectively. Finally, xenograft and metastatic mouse models using MDA231 cells were established to confirm the effect of BRD7 on tumor growth and metastasis.

**Results:**

Here, the results of a series of assays in vitro indicated that BRD7 has the ability to inhibit the mobility, migration and invasion of breast cancer cells. In addition, YB1 was identified as a novel interacting protein of BRD7, and BRD7 was found to associate with the C-terminus of YB1 via its N-terminus. BRD7 decreases the expression of YB1 through negatively regulating YB1 phosphorylation at Ser102, thereby promoting its proteasomal degradation. Furthermore, gene set enrichment analysis revealed that epithelial-mesenchymal transition (EMT) is the common change occurring with altered expression of either BRD7 or YB1 and that BRD7 represses mesenchymal genes and activates epithelial genes. Moreover, restoring the expression of YB1 antagonized the inhibitory effect of BRD7 on tumorigenicity, EMT, invasiveness and metastasis through a series of in vitro and in vivo experiments. Additionally, BRD7 expression was negatively correlated with the level of YB1 in breast cancer patients. The combination of low BRD7 and high YB1 expression was significantly associated with poor prognosis, distant metastasis and advanced TNM stage.

**Conclusions:**

Collectively, these findings uncover that BRD7 blocks tumor growth, migration and metastasis by negatively regulating YB1-induced EMT, providing new insights into the mechanism by which BRD7 contributes to the progression and metastasis of breast cancer.

## Background

Epithelial-mesenchymal transition (EMT) is an initially reversible biological process and plays an important role in tumor development; during this process, epithelial cells gradually lose their adhesion to each other, which not only reshapes their polarity and cytoskeleton but also increases their proliferative, migratory and invasive abilities, enhances their apoptosis resistance, and promotes their acquisition of stem cell characteristics [1]. During EMT, rapid morphological changes, including loss of the epithelial phenotypes and acquisition of mesenchymal phenotypes, occur in cells. In addition, EMT reprograms gene expression, downregulating epithelial genes and upregulating mesenchymal genes. For instance, E-cadherin levels are decreased, resulting in enhanced invasion and metastasis, and vimentin and N-cadherin levels are increased. Loss of E-cadherin expression has been considered the most significant feature of EMT. Moreover, a series of transcription factors, including Snail, slug, ZEB1 and twist, are involved in the regulation of EMT [2].

As a member of the SWI/SNF complex, BRD7 is a potential transcription factor and was first cloned in the early stage of our research [3]. BRD7 is usually under expressed and plays a role as a tumor suppressor in many malignant tumors; in addition, it is associated with advanced disease and poor prognosis in cancers such as nasopharyngeal cancer (NPC), breast cancer, ovarian cancer, lung cancer and liver cancer [4–6]. Immunohistochemistry (IHC) of breast cancer and normal tissues confirmed that the low expression and mainly nuclear localization of BRD7 is present in tumor tissues and a high level of BRD7 is viewed as a positive prognostic factor [7, 8]. BRD7 participates in a myriad of cellular processes, including proliferation inhibition, cell cycle arrest, apoptosis induction, migration and invasion inhibition, embryonic death [6, 9]. In the early stage of inflammation, BRD7 inhibits the activation of the NF-κB pathway and the occurrence of inflammation by inhibiting the expression and activity of IL-6, TNF-a, p65, CXCL-1 and iNOS [10]. It is pointed out in the literature that BRD7-deficient tumor cells exhibit increased sensitivity to interferon- γ, promote the activation of effector T cells and kill tumor cells [11], suggesting that BRD7 may be a very promising target for tumor immunotherapy. Therefore, studying the molecular mechanism of BRD7 in tumorigenesis has substantial value for clinical application.

It has since been established that BRD7 delays tumor progression by negatively regulating the PI3K/AKT, P53, Ras-Raf-MEK-ERK and β-catenin pathways [5, 12–14]. A recent study showed that BRD7 is the target gene of miR300 and that overexpression of BRD7 can antagonize the promotive effect of miR300 on cell growth and invasion [15]. In addition, in ovarian cancer, BRD7 is expressed at a low level, inhibits tumor growth and invasion and potently accelerates the apoptosis of ovarian cancer cells through inhibition of the nuclear entry of β-catenin in a p53-independent manner [16]. These studies indicate that BRD7 exerts an inhibitory effect on tumor invasion and metastasis in some types of tumors. Y-box binding protein-1 (YB1), a DNA/RNA-binding protein containing a conserved cold shock domain (CSD), is commonly overexpressed and associated with poor clinical outcomes in a broad range of human carcinomas, including breast cancer, liver cancer and lung cancer [17]. The YB1 transgenic mouse induces chromosomal instability leading to the development of breast cancer with an incidence of 100% [18]. An increasing number of studies have revealed that YB1, a transcriptional activator, induces tumor growth, invasion and metastasis at the transcriptional level in the nucleus and at the translational level in the cytoplasm [19]. Notably, YB1 is reported to facilitate EMT of tumor cells at both the transcriptional and translational levels and can be degraded by the ubiquitin proteasome pathway. And a high level of YB1 exists in breast cancer and is significantly associated with poor overall survival and distant metastasis [20, 21].

As an important tumor suppressor gene, BRD7 plays an anti-tumor role in breast cancer. However, the roles of BRD7, its association with YB1, and the molecular mechanism by which it is involved in tumor invasion and metastasis in breast cancer are not well understood and remain to be determined. Therefore, our aim was to gain insight into the function and molecular biological mechanism of BRD7 involved in breast cancer growth, invasion and metastasis. In this report, we demonstrated that BRD7 inhibits tumor growth, migration and invasion in breast cancer both in vitro and in vivo. BRD7 interacted with YB1 and facilitated the ubiquitin-mediated proteasomal degradation of YB1, which is dependent on the phosphorylation level of YB1 at the S102 site. Furthermore, a series of rescue experiments confirmed that BRD7 blocks tumor growth, EMT and metastasis through a YB1-mediated malignant phenotype. Importantly, combined with the results of our previous studies [8], clinical data analysis revealed that BRD7 is negatively correlated with YB1 and that low expression of BRD7 combined with high expression of YB1 is an effective marker for poor prognosis and is associated with tumor size, distant metastasis and advanced TNM stage in breast cancer patients.

## Methods

### Cell culture and virus packaging

MDA-MB-231 (MDA231), MCF7 and HEK293T cells were obtained from ATCC (The Global Bioresource Center). MCF7 cells were cultured in Roswell Park Memorial Institute 1640 (RPMI1640, Life Technologies, USA) medium supplemented with 10% fetal bovine serum (FBS). MDA231 and HEK293T cells were routinely cultured in Dulbecco’s modified Eagle’s medium (DMEM, Life Technologies, USA) containing 10% FBS. Transfection was performed with Lipofectamine 3000 according to the manufacturer’s protocols (Invitrogen, USA), as described previously [12]. The BRD7-overexpressing and BRD7-shRNA cells were generated by lentiviral infection. BRD7-overexpressing lentivirus was purchased from GenePharma (Suzhou, China), YB1-overexpressing lentivirus was obtained using a YB1 expression plasmid purchased from Sino biological (Beijing, China) and packaged in HEK293T cells, and BRD7 shRNA lentivirus was obtained using the expression vector pLVTH/shBRD7. The BRD7 siRNA sequence was 5′-GUGCCAAGAUUAUCCGUAUdTdT-3′. A total of 10 μg of the corresponding expression vector and 7.5 μg of the packaging vectors (pMD2G and pSPAX2) were co-transfected into HEK293T cells for 48 h. The virus-containing supernatant was collected, centrifuged at 2000 rpm for 10 min and filtered through a 0.22 μm membrane. Tumor cells were infected with the supernatant for 48 h and screened for 72 h with 2 μg/ml puromycin in DMEM.

### Clinical data information

 A total of 220 breast cancer and 43 normal breast paraffin-embedded samples were collected from the Second Xiangya Hospital of Central South University from  November 2001 to September 2012, and this study was approved by Ethics Review Committees/Institutional Review Boards of Central South University.Clinicopathologic features of the breast cancer patients mainly included gender, age, tumor size, node metastasis, distant metastasis, clinical tumor node metastasis (TNM) stage, pathology diagnose, survival time and molecular subtype. The immunohistochemical scores of clinical samples were based on the detailed procedures described in other articles [8].

### RNA extraction and qRT-PCR

Total RNA was extracted from MDA231 and MCF7 cells with TRIzol Reagent (15596–026, Invitrogen, USA). First strand cDNA synthesis with 2 μg of total RNAs was performed using a RevertAid first strand cDNA synthesis Kit according to the instructions (K1622, Thermo Scientific, USA). Detailed experimental procedures are referred to our published literature [22]. The gene expression was monitored using fluorescence quantitative PCR (CFX96, Bio-Rad, USA). Primer sequences used in this article are listed in Table 1.

**Table 1:** Primer sequences in this paper

Name	sequences	Primer Length (bp)
YB1-S102A-Forward	CCCCAGGAAGTACCTTCGCGCTGTAGGAGATGGAGAGACTGTGGAGT	47
YB1-S102A-Reverse	ACTCCACAGTCTCTCCATCTCCTACAGCGCGAAGGTACTTCCTGGGG	47
E-cadherin- Forward	TGCCCAGAAAATGAAAAAGG	20
E-cadherin -Reverse	GTGTATGTGGCAATGCGTTC	20
vimentin- Forward	CCTGAACCTGAGGGAAACTAA	21
vimentin-Reverse	GCAGAAAGGCACTTGAAAGC	20
Snail1- Forward	TGCGTCTGCGGAACCTG	17
Snail1-Reverse	GGACTCTTGGTGCTTGTGGA	20
Claudin1-Forward	CTGTCATTGGGGGTGCGATA	20
Claudin1-Reverse	CTGGCATTGACTGGGGTCAT	20
BRD7-Forward	AAGCACACGCCTTCAAGAGT	20
BRD7- Reverse	TTCCTTCACGATGCGGTCAA	20
YB1-Forward	AAGGAGAAAAGGGTGCGGAG	20
YB1-Reverse	CCTACGACGTGGATAGCGTC	20
GAPDH-Forward	CAACGGATTTGGTCGTATTGG	21
GAPDH-Reverse	TGACGGTGCCATGGAATT	19

### Cell proliferation experiment

MDA231 cells (600 cells / well) and MCF7 cells (1000 cells / well) separately were plated into 96 - well plates in 200 μL complete medium and further incubated for different periods (0, 1, 2, 3, 4 d). At different time points, 20 μL CCK8 (B34302, Bimake, USA) was added to each hole for further incubation for 3 h, and the absorbance value was determined at 450 nm by the microplate analyzer.

### Wound healing and matrigel invasion assays

For wound healing assays, MDA231 or MCF7 cells were seeded in 6 - wells plates and cultured in routine condition, and 10 μL tips were used for the wound healing assays when the cell density was above 95%. Then the cells were washed once with D-hanks and cultured with a low concentration of serum. Photos were taken at different time points (0, 24, 36 and 48 h) and statistically analyzed by Image J.

For Matrigel invasion assays, MDA231 or MCF7 cells suspended in 200 μL serum-free medium were implanted in transwell chambers covered with 10% matrigel (BD, Franklin Lakes, NJ, USA). When appropriate cells were filtered to the bottom of the chamber, the cells were fixed with 4% paraformaldehyde and stained with crystal violet. Five random fields per group were photographed under an optical microscope and the number of cells were counted.

### Three-dimensional invasion assay

The experimental procedures were conducted with reference to the methods in previously published papers [23, 24]. Approximately 100 μL of Matrigel was spread on the bottom of a 24 - well plate for 2 ~ 4 h at 37 °C until the colloid solidified. MDA231 cells were collected at a density of 10,000 cells per ml in medium containing 10% Matrigel. Then, 200 μL of the cell suspension was added to the previously coagulated gel and cultured at 37 °C for 1 h. Then, 200 μL of complete medium containing 10% FBS was added, and the cells were cultured until the appropriate time points. Clonal spheroids were observed and photographed under a microscope. According to statistical methods used in a previous study [24], clonal spheroids were divided into two types based on the cell protrusions: cells with distinct protrusions were considered invasive clonal spheroids, and other cells were considered noninvasive.

### Immunofluorescence assay

MCF7, MDA231 and HEK293T cells were co-transfected with flag-BRD7 and HA-YB1 expression plasmids for 48 h, respectively. Then cells were washed three times with PBS and incubated with 4% paraformaldehyde for 1 h at room temperature, and then the cells were permeabilized with 0.3% Triton X-100 (DH351–5, Genview, china) for 30 min, inactivated with 0.3% H_2_O_2_ for 30 min then blocked for 30 min in normal goat serum (AR0009, BOSTER Biological Technology) and followed by incubation with primary antibody overnight at 4 °C. Then the cells were incubated with relative secondary fluorochrome-labelled antibodies for 1 h at 37 °C, and followed by incubation with DAPI (Beyotime Institute of Biotechnology, china) for 1 min at room temperature to stain the nuclei. Cellular fluorescence was monitored using immunofluorescence microscope (Leica, USA).

### Western blotting

Briefly, 1 × 10^6^ cells including MDA231, MCF7 and HEK293T were separately collected into microcentrifuge tubes and lysed in Western and IP lysates buffer (P0013, Beyotime Biotechnology, china) supplied with protease inhibitors and phosphatase inhibitors (Roche, USA) on ice for 30 min and vigorously vortexed every 10 min and followed by high-speed centrifuge for 15 min. The supernatant cytosolic fractions were collected into another microcentrifuge tubes. Protein concentration was determined by the bicinchoninic acid (BCA) method using Pierce™ BCA Protein Assay Kit (23227, Thermo Fisher, USA) according to the manufacturer’s instructions. Fifty micrograms protein samples were then denatured in 1 × SDS-page protein loading buffer (P0015, Beyotime Institute of Biotechnology, china) at 95 °C for 5 min. Protein were separated by 10% SDS–PAGE and transferred to PVDF membranes (ISEQ00010, Millipore, USA). The primary antibody was incubated overnight, and the second antibody was incubated at 37 °C for 1 h. Primary antibodies used in western blotting are as follow. Antibodies against anti-BRD7 (51009–2-AP, proteintech, 1:1000 dilution), anti-YB1 (CY5462, Abways Technology, 1:1000 dilution), anti-Phospho-YB1 (Ser102) Antibody (CSB-PA204680, Cusabio, 1:1000 dilution), anti-HA (561–7, MBL, 1:1000 dilution), anti-Flag (F3040, Sigma-Aldrich, 1:1000 dilution), anti-Vimentin (ARG66302, arigo, 1:1000 dilution, 1:200 dilution for IF), anti-Snail (C15D3, CST, 1:1000 dilution), anti-E-cadherin (24E10, CST, 1:1000 dilution), anti-Claudin1 (D5H1D, CST, 1:1000 dilution, 1:50 dilution for IF) and anti-GAPDH (10494–1-AP, proteintech, 1:20000 dilution). Secondary antibodies used in western blot are HRP-conjugated Affinipure Goat Anti-Mouse IgG(H + L) (1SA00001–1, proteintech, 1:20000 dilution) and HRP-conjugated Affinipure Goat Anti-Rabbit IgG(H + L) (SA00001–2, 1:20000 dilution). Bands are obtained by Western Blotting Substrate (32106. Pierce™ ECL Western Blotting Substrate, Thermo Scientific, USA), and captured by chemiluminescence imaging systems (MiniChemi™ I, SAGECREATION, china).

### Co-immunoprecipitation

MDA231, MCF7 and HEK293T cells were co-transfected with BRD7 and YB1 expression plasmids for 48 h, respectively. Whole protein was extracted by Western and IP lysates buffer as described above. Protein A/G beads (B23202, Protein A/G immunoprecipitation magnetic beads, Bimake, USA) were first incubated with indicated antibodies for 2 h at RT. Protein fractions (2 mg) and Protein A/G beads were incubated overnights at 4 °C. Beads that contained affinity-bound proteins were then washed five times with Western and IP lysates buffer, and denatured in 30 μL 2 × SDS loading buffer at 95 °C for 5 min. Finally, the sample was placed on ice for follow-up work or stored at − 80 °C.

### Co-immunoprecipitation and mass spectrometry analysis (Co-IP-MS)

HEK293T cells were transfected with the plasmids pIRES2-EGFP-BRD7/3Flag for 48 h using Lipofectamine 3000 according to the manufacturer’s protocols (Invitrogen), and the protein extracts were incubated with Protein A/G beads conjugated to anti-flag or anti-IgG antibodies overnight according to the above coimmunoprecipitation assay procedure. Then, the samples were denatured in 30 μL of 2× SDS loading buffer at 95 °C for 5 min and resolved by 10% SDS–PAGE. Following protein separation, the gel was stained using a Coomassie blue staining kit (P0017A, Beyotime Biotechnology, China) and shaken gently overnight in double distilled water for decolorization. The bands were cut into tiny micelles, decolorized to transparency with decoloring solution (50% Acetonitrile (ACN) and 25 mM NH_4_HCO_3_), and infiltrated with 250 μL of protein protection solution (55 mM IAA and 25 mM NH_4_HCO_3_) at room temperature for 30 min. The samples were further infiltrated with 250 μL of a protective solution (25 mM dithiothreitol (DTT) and 25 mM NH_4_HCO_3_) at room temperature for 30 min, dehydrated with 100% ACN and dried using a vacuum drier; then, an appropriate amount of trypsin was added for digestion at 37 °C overnight. The samples were dehydrated with solution buffer (0.1% trifluoroacetic acid and 70% ACN). Then, the peptides were further diluted with 0.1% formic acid and was analyzed by nano-LC-MS/MS using an LTQ Velos Orbitrap MS (Thermo Fisher Scientific, Waltham, MA, USA) coupled with an UltiMate RSLCnano LC system (Dionex, Sunnyvale, CA, USA) [24].

### In vivo ubiquitination assay

For the total ubiquitination assay, MDA231 cells were co-transfected with flag-BRD7, HA-YB1 and HA-Ub for 36 h, treated with 20 μM MG132 for 4 h and then lysed in Western and IP lysis buffer supplemented with protease inhibitors. Immunoprecipitation was conducted using anti-YB1 antibodies. Western blotting was used to detect the expression of YB1, Ub and flag/BRD7.

For the exogenous ubiquitination assay, HEK293T cells were co-transfected with HA-BRD7 and flag-YB1 of wild-type (flag-YB1) or YB1 mutant (flag-YB1S102A) plus HA-Ub for 36 h, treated with 20 μM MG132 for 4 h and lysed in Western and IP lysis buffer supplemented with protease inhibitors and phosphatase inhibitors. Immunoprecipitation was performed using anti-flag antibodies. Western blotting was further performed to detect the expression of Ub, flag, p-YB1 S102A and HA.

### RNA sequencing and data analysis

Total RNA was isolated from MDA231 cells ectopically expressing BRD7 and the corresponding control. The results of analysis with the Agilent 2100 system showed that the RNA quality completely met the requirement for Illumina HiSeq™ 4000 sequencing (Lnc-seq). Filtering, quality assessment, comparative analysis and gene annotation were performed on the sequencing data by Genedenovo Biotechnology Co., Ltd. (Guangzhou, China). The mRNA gene expression data of MDA231 cells with BRD7-overexpressing and control were performed in the current study can be obtained from the Sociedad Rural Argentina (SRA) database (https://www.ncbi.nlm.nih.gov/sra) under accession number PRJNA562788.

Datasets GSE60964 and GSE6562 were downloaded from the NCBI Gene Expression Omnibus (GEO, http://www.ncbi.nlm.nih.gov/geo) database. These three datasets were subjected to gene set enrichment analysis (GSEA) performed with GSEA 2.09. The mRNA expression data (GSE60964 and GSE6562) were divided into two groups according to the expression level of YB1. The BRD7 data sets of BRD7 were divided into two groups including BRD7 overexpression and control group. In addition, we analyzed the expression of YB1 in breast cancer of TCGA data in UALCAN (http://ualcan.path.uab.edu/) [25]. We also analyzed the association of YB1 expression with survival in breast cancer via Kaplan-Meier Plotter (http://kmplot.com/analysis/) [26].

### Immunohistochemistry (IHC) and hematoxylin and eosin (H&E) staining

After tumor tissue was taken, fixed and embedded with the paraffin and sectioned, the sections were dewaxed in xylene and rehydrated using graded concentrations of ethanol and distilled water. For HE assays, the sections were directly stained with hematoxylin-eosin. For IHC experiments, the procedures are described in the previous published article [10]. Briefly, the primary antibody was incubated overnight at 4 °C and the second antibody was incubated at room temperature for 30 min. The primary antibodies used in this article includes anti-YB1(#4202, CST, 1:50 dilution), anti-BRD7 antibody (51009–2-AP, proteintech, 1:500 dilution), anti-Ki67 antibody (ZA0502, ZSGB-BIO), anti-E-cadherin antibody (#24E10, CST, 1:100dilution) and anti-Vimentin antibody (ARG66302, arigo,1:500).

### Mouse model

Five-week-old female BALB/c nude mice were purchased from CAVENS (Jiangsu, China) and fed in the SPF level barrier system of the laboratory animal science department of Central South University. Animal experiments were divided into three groups: the control, BRD7 overexpression and BRD7 overexpression with simultaneous YB1 overexpression (YB1 restoration) groups. For the breast cancer xenograft model (*n* = 5 per group), 3 × 10^6^ MDA231 cells in 100 μL of saline were subcutaneously inoculated into the left shoulders of 5-week-old female nude mice. Tumor size was observed and measured every 4 days. Tumor volume was evaluated using the following formula: volume = (length × width^2^) × 1/2. All mice were sacrificed 29 days after subcutaneous inoculation, and the tumors were surgically collected, fixed with formalin and embedded in paraffin for IHC. For the metastatic model (*n* = 11 per group), 2 × 10^6^ MDA231 cells in 200 μL of saline were injected into the tail vein of nude mice. Thirty-one days post transplantation, all mice were sacrificed, and lung tissues were isolated and embedded in paraffin for H&E staining.

### Statistical analysis

The relationships between the BRD7 and YB1 expression levels and clinicopathological characteristics of patients with breast cancer were assessed using the chi-squared test. Spearman’s rank correlation coefficient was used to assess the significance of the association between BRD7 and YB1 expression in breast cancer. Kaplan-Meier analysis was performed to generate OS curves, and statistical significance was assessed using the log-rank test. Comparisons between two groups of data were analyzed using Student’s t-test, and multiple sets of data were analyzed with one-way ANOVA; data are presented as the means ± SDs or means ± SEMs using GraphPad Prism 8.01. *P* values less than 0.05 indicates statistical significance (ns, *p* > 0.05; *, *p* < 0.05; **, *p* < 0.01; and ***, *p* < 0.001).

## Results

### High expression of BRD7 prohibits breast cancer cell growth and invasion in vitro

**Fig. 1:**
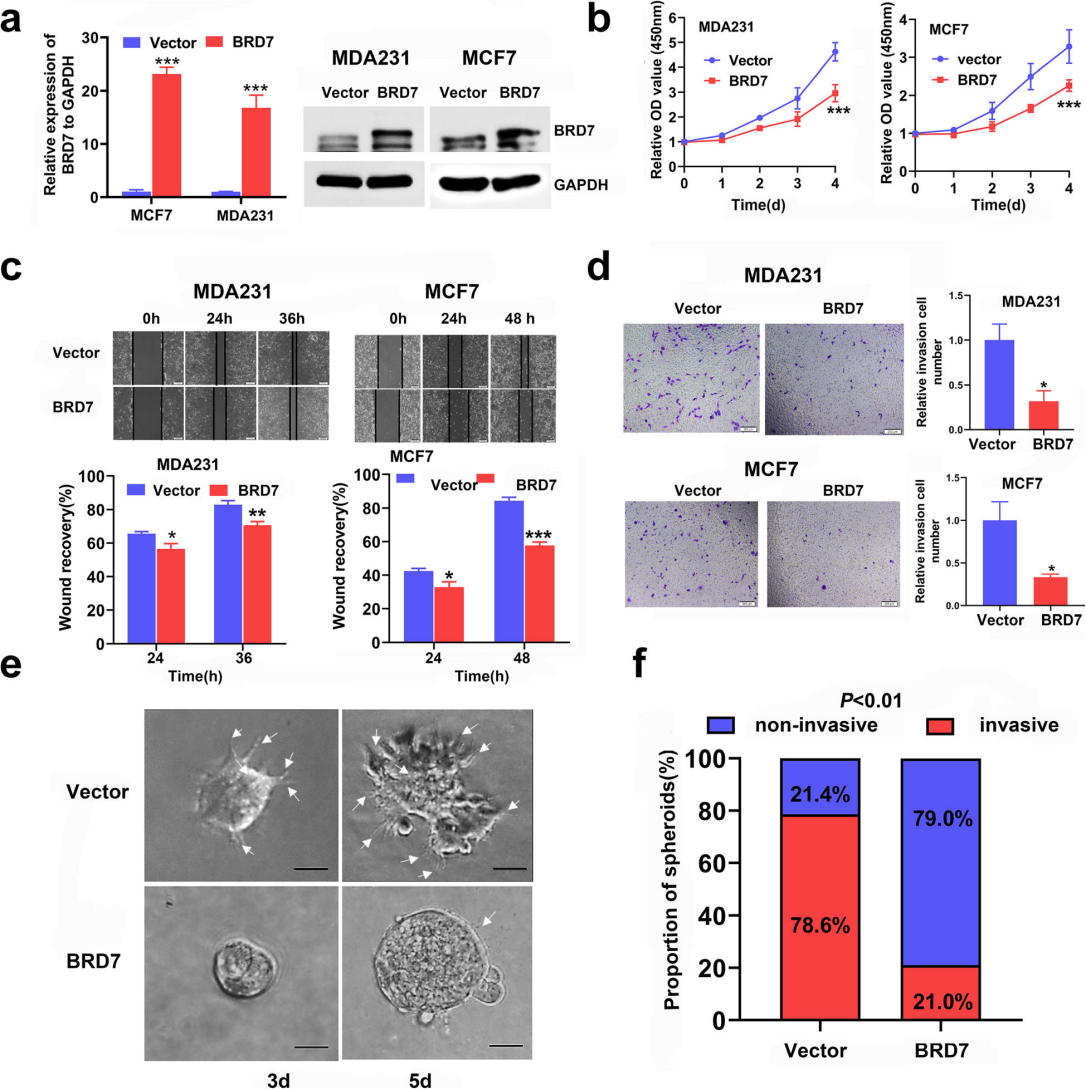
BRD7 inhibits cell migration and invasion in breast cancer cells. **a** qPCR and western blotting analysis of BRD7 expression in MDA231 and MCF7 cells stably transfected with BRD7 expression plasmid or control. Data represent means ± SDs. ***, *p* < 0.001. **b** CCK8 analysis of cell proliferation in MDA231 and MCF7 cells stably transfected with BRD7 expression plasmid or control. Data represent means ± SDs. ***, *p* < 0.001. **c** Scratch wound healing analysis of cell migration in MDA231 and MCF7 cells stably transfected with BRD7 expression plasmid or control. Quantification of wound recovery rate of the two group. Data represent means ± SEMs. *, *p* < 0.05, **, *p* < 0.01, ***, *p* < 0.001. **d** Matrigel invasion analysis of cell invasive capabilities in MDA231 and MCF7 cells stably transfected with BRD7 expression plasmid or control. Data represent means ± SEMs. *, *p* < 0.05. **e** Three-dimensional invasion analysis of cell invasive capabilities in MDA231 cells stably transfected with BRD7 expression plasmid or control. The white arrows represent prominent protrusions, scale bar, 50 μm. **f** Quantification of invasive and non-invasive clonal spheroids in BRD7 overexpression and control group. Data represent means ± SDs

To explore the role of BRD7 in breast cancer, two stable cell lines with BRD7 overexpression and one stable cell line with BRD7 interference via shRNA-mediated depletion of BRD7 were established. We initially examined the overexpression and interference effect of BRD7 in two cell lines using qPCR and WB. The results showed that BRD7 was successfully overexpressed in MDA231 and MCF7 cells and knocked down in MDA231 cells (Fig. 1a and Additional file 1: Figure S1a). Importantly, the results of CCK8 assays showed that BRD7 overexpression significantly inhibited the growth of breast cancer cells compared with control cells (Fig. 1b). Next, the scratch wound healing assay results revealed that BRD7 overexpression significantly suppressed cell migration, while BRD7 silencing produced the opposite effect (Fig. 1c and Additional file 1: Figure S1b). The Matrigel invasion assay results showed that the invasive capability was significantly impaired by forced expression of BRD7 in MDA231 and MCF7 cells and enhanced by BRD7 knockdown in MDA231 cells (Fig. 1d and Additional file 1: Figure S1c). Considering the significant effects of BRD7 on cell proliferation, migration and invasion, we further detected the changes in cell mobility and invasion after BRD7 alteration using three-dimensional invasion assays. Amazingly, the number of spherical clones of invasive cells and prominent protrusions at the edges of the cells were significantly reduced following overexpression of BRD7, while BRD7 knockdown appreciably produced the opposite effect (Fig. 1e and f and Additional file 1: Figure S1d and e). Overall, these results demonstrated that BRD7 inhibits the invasion and metastasis of breast cancer cells. These data support the idea that BRD7 plays an essential role in regulating breast cancer growth and metastasis.

### BRD7 binds the C-terminus of YB1 via its N-terminus

**Fig. 2:**
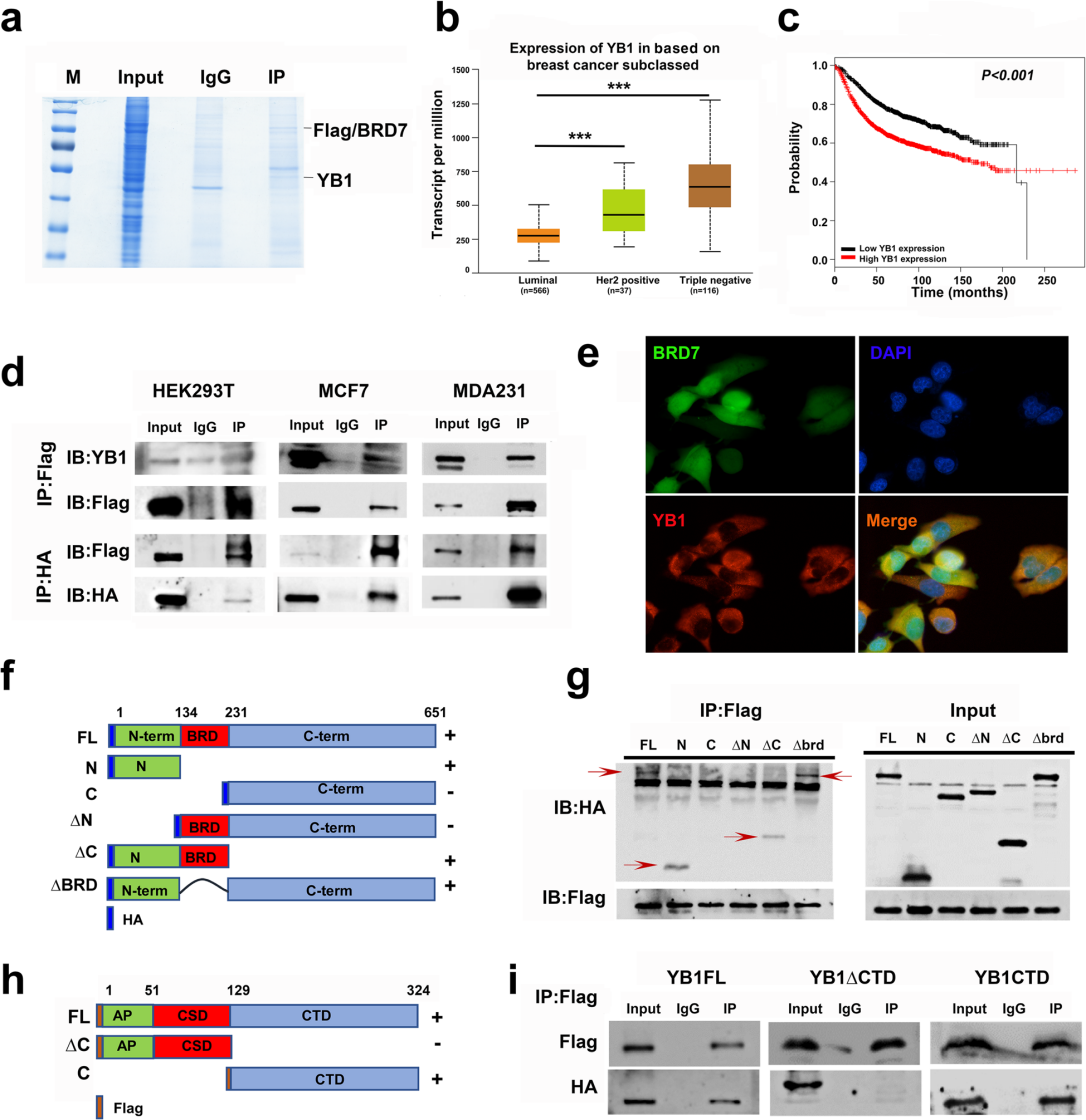
BRD7 interacts with YB1. **a** Coomassie blue staining of co-immunoprecipitation using anti-IgG or anti-flag antibodies in BRD7 overexpression HEK293T cells. **b** Quantification of YB1 expression in TCGA BRCA database (*n* = 823) of different clinical types (Luminal, her2 positive and triple negative types). **c** Km-plot analysis of YB1 expression and survival of breast cancer patients consist of 1976 patients in YB1 low expression group and 1975 patients in YB1 high expression group. **d** Co-immunoprecipitation (top) using anti-flag antibodies in flag-BRD7 overexpressed of HEK293T, MDA231 and MCF7 cells and western blotting analysis of flag and YB1. Co-immunoprecipitation (down) using anti-HA antibodies in HEK293T, MDA231 and MCF7 cells of flag-BRD7 and HA-YB1 overexpression and western blotting analysis of HA and flag. **e** IF using anti-flag or anti-YB1 antibodies in MDA231 cells of flag-BRD7 overexpression. **f** Schematic illustration of different extents of brd7 mutants. **g** Co-immunoprecipitation using anti-flag antibodies in HEK293T cells co-transfected with HA-BRD7 deletion mutants and flag-YB1 and western blotting analysis of HA and flag. **h** Schematic illustration of different extents of YB1 mutants. **i** Co-immunoprecipitation using anti-flag antibodies in HEK293T cells co-transfected with flag-YB1 deletion mutants and HA-BRD7. Western blotting analysis of flag and HA

To examine the molecular mechanism by which BRD7 inhibits proliferation and metastasis in breast cancer, the interacting proteins of BRD7 were screened in HEK293T cells overexpressing BRD7 via coimmunoprecipitation combined with mass spectrometry after gel staining with Coomassie blue (Fig. 2a). The interacting proteins were ranked according to the scores, and YB1 was one of the top 20 molecules and is a vital oncogene in an assortment of tumors such as breast cancer, colorectal cancer and lung cancer [27]. Through analysis of public data sets in the UALCAN cancer database, we found that YB1 had the highest expression in samples from TNBC, the most aggressive form of breast cancer, followed by HER2-positive breast cancer, and had the lowest expression in luminal-type breast cancer in the TCGA data set (Fig. 2b). Moreover, patients with high expression of YB1 had poor prognosis (Fig. 2c). This finding suggests that YB1, an important transcription factor, is one of the potential interacting molecules of BRD7.

To determine whether there is an interaction between BRD7 and YB1, co-IP experiments were carried out in HEK293T, MDA231 and MCF7 cells. It is interesting to note that YB1 was clearly present in BRD7-immunoprecipitated complexes but not in the IgG-immunoprecipitated complexes, suggesting a tight interaction between BRD7 and YB1 (Fig. 2d, top). And these results were further conformed by the immunofluorescence experiments, which showed that BRD7 was colocalized with YB1 mainly in the cytoplasm of MDA231, MCF7 and HEK293T cells (Fig. 2e and Additional file 1: Figure S2). Therefore, these results strongly support the idea that there is an interaction between BRD7 and YB1.

To characterize the binding domain that forms the basis for the interaction between BRD7 and YB1, we first constructed a series of BRD7 deletion mutants, as shown in the schematic diagram (Fig. 2f). The results of pulldown assays showed that YB1 interacts with wild-type BRD7, the N-terminal domain mutant (1–134 aa), the ∆C-terminal domain mutant (∆232–651 aa) and the ∆BRD domain mutant (∆135–231 aa) but not with the C-terminal domain mutant (232–651 aa) or the ∆N-terminal domain mutant (∆1–134 aa), suggesting that the interaction of BRD7 with YB1 is dependent on the N-terminal domain of BRD7 (Fig. 2g). Previous studies confirmed that YB1 consists of an AP domain (1–51 aa), a highly conserved cold shock domain (CSD, 52–129 aa) and a C-terminal domain (CTD, 130–324 aa) and the binding sites of YB1 and numerous proteins are located in the CTD of YB1 [19]. Thus, we constructed the YB1-∆CTD (1–129 amino acids) and CTD (130–324 aa) mutants (Fig. 2h). HEK293T cells were co-transfected with HA-BRD7 plus wild-type flag-YB1 or YB1 deletion mutants (CTD and ∆CTD) for 48 h, and co-immunoprecipitation was subsequently performed. The results demonstrated that BRD7 could interact with wild-type YB1 and the YB1 CTD mutant but not with the YB1 ∆CTD mutant (Fig. 2i). These findings indicate that the N-terminal domain of BRD7 binds to the carboxyl terminus of YB1.

### High expression of BRD7 induces ubiquitin-mediated degradation of YB1 dependent on YB1 Ser102 phosphorylation

**Fig. 3:**
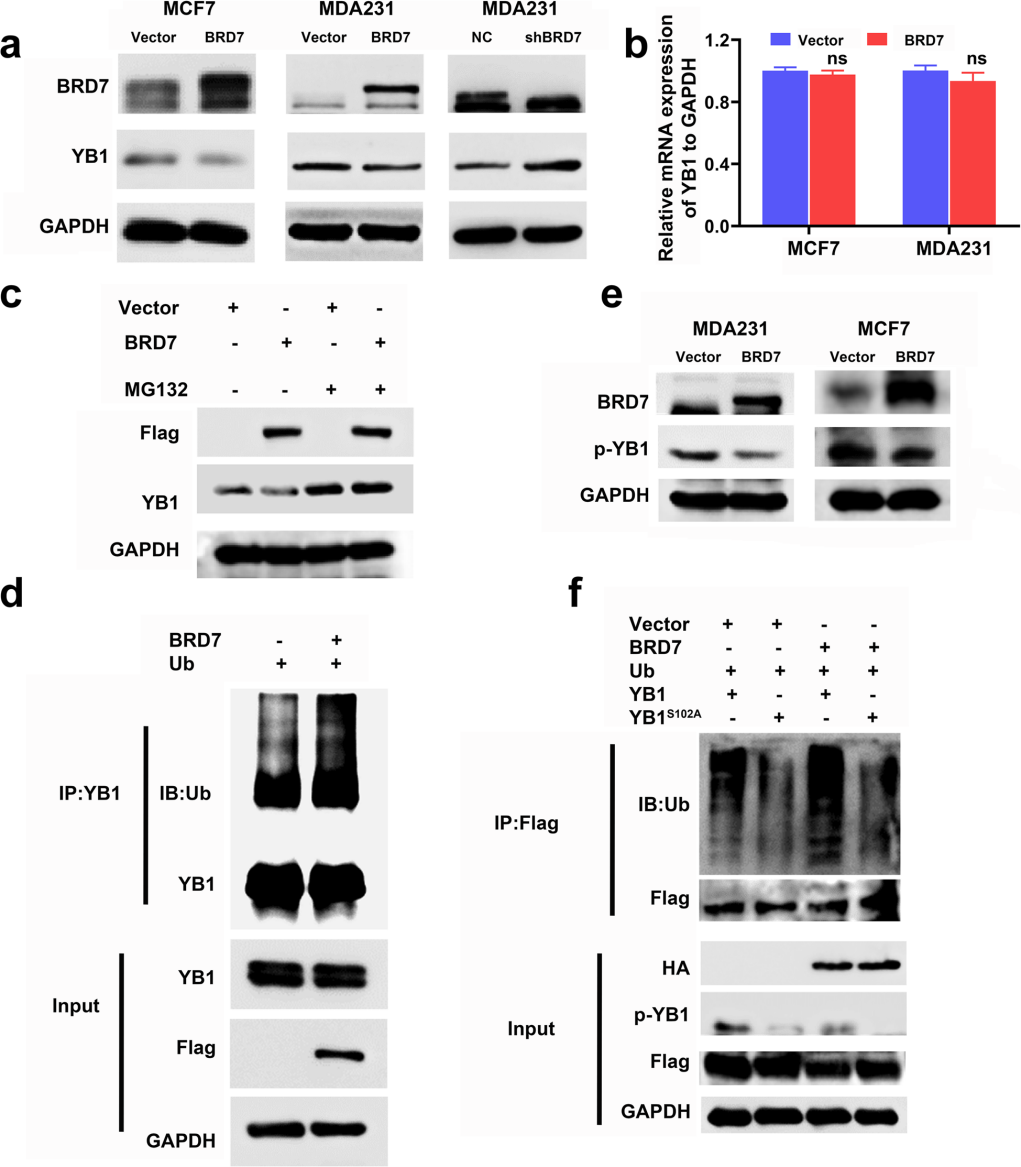
BRD7 induces ubiquitination degradation of YB1 depended on YB1 Ser102 phosphorylation. **a** Western blotting analysis of BRD7 and YB1 in MDA231 and MCF7 cells with BRD7 overexpression. **b** qPCR analysis of YB1 in BRD7 overexpression of MDA231 and MCF7 cells. **c** Western blotting analysis of Flag-BRD7 and YB1 in BRD7 overexpressed MDA231 cells treated with or without MG132 (20 μM) for 4 h. **d** Co-immunoprecipitation using anti-YB1 antibodies in MDA231 cells co-transfected Ub with flag-BRD7 or control and treated with or without MG132(20 μM) for 4 h. Western blotting analysis of Ub and YB1. **e** Western blotting analysis of BRD7 and p-YB1ser^102^ in MDA231 and MCF7 cells transfected with BRD7. **f** Co-immunoprecipitation using anti-flag antibodies in HEK293T cells co-transfected by BRD7 along with either YB1 wild-type or YB1 mutant plus HA-ubiquitin for 48 h, treated with MG132(20 μM) for 4 h. Western blotting analysis of Ub, flag, HA, p-YB1 and GAPDH

Furthermore, we found that the YB1 protein level was clearly decreased in BRD7-overexpressing cells while increased in BRD7-knockdown cells, but YB1 mRNA level has no significant change after BRD7 overexpression (Fig. 3a and b). In addition, the expression of BRD7 mRNA and protein was detected in YB1-overexpressing cells. These results showed that the mRNA and protein expression levels of BRD7 were not significantly altered after YB1 overexpression in MCF7 cells (Additional file 1: Figure S3a and b), indicating that a unidirectional regulatory relationship exists between BRD7 and YB1- that is, BRD7 negatively regulates YB1 but YB1 has no effect on the expression of BRD7. Collectively, these results suggest that BRD7 might regulate the proliferation and migration of breast cancer cells by regulating YB1 at the posttranslational level. Furthermore, we treated BRD7-overexpressing cell lines with the proteasome inhibitor MG132 for 4 h. As a result, the decreased YB1 expression under BRD7 overexpression was abolished by treatment with MG132 (Fig. 3c). More accumulation of YB1 ubiquitination was observed in the BRD7 overexpression group than in the control group (Fig. 3d), suggesting that BRD7 induces the ubiquitination-mediated degradation of YB1.

YB1 contains a conserved phosphorylation site at Ser102; phosphorylation at this site can be activated by the PI3K/AKT and MAPK signaling pathways and carries out important functions in tumor progression [28–31]. Subsequently, we examined whether BRD7 influences the phosphorylation level of YB1. Strikingly, overexpression of BRD7 profoundly decreased the phosphorylation level of YB1 S102 in both MDA231 and MCF7 cells, as shown in Fig. 3e. Given that the phosphorylation level of YB1 is closely related to the ability of YB1 to function, we propose a hypothesis that the ubiquitination degradation of YB1 is dependent on its phosphorylation level. As a result, ectopic expression of BRD7 obviously induced the ubiquitination-mediated degradation of YB1, whereas this cumulative effect was significantly impaired when the Ser102 phosphorylation site on YB1 was mutated to Ala using the point mutation technique (Fig. 3f). Based on these results, we concluded that the N-terminal domain of BRD7 interacts with the C-terminus of YB1 and that BRD7 has a promotive effect on YB1 degradation, which is dependent on YB1 phosphorylation at Ser102.

### High level of BRD7 inhibits the process of epithelial-mesenchymal transition in breast cancer cells

**Fig. 4:**
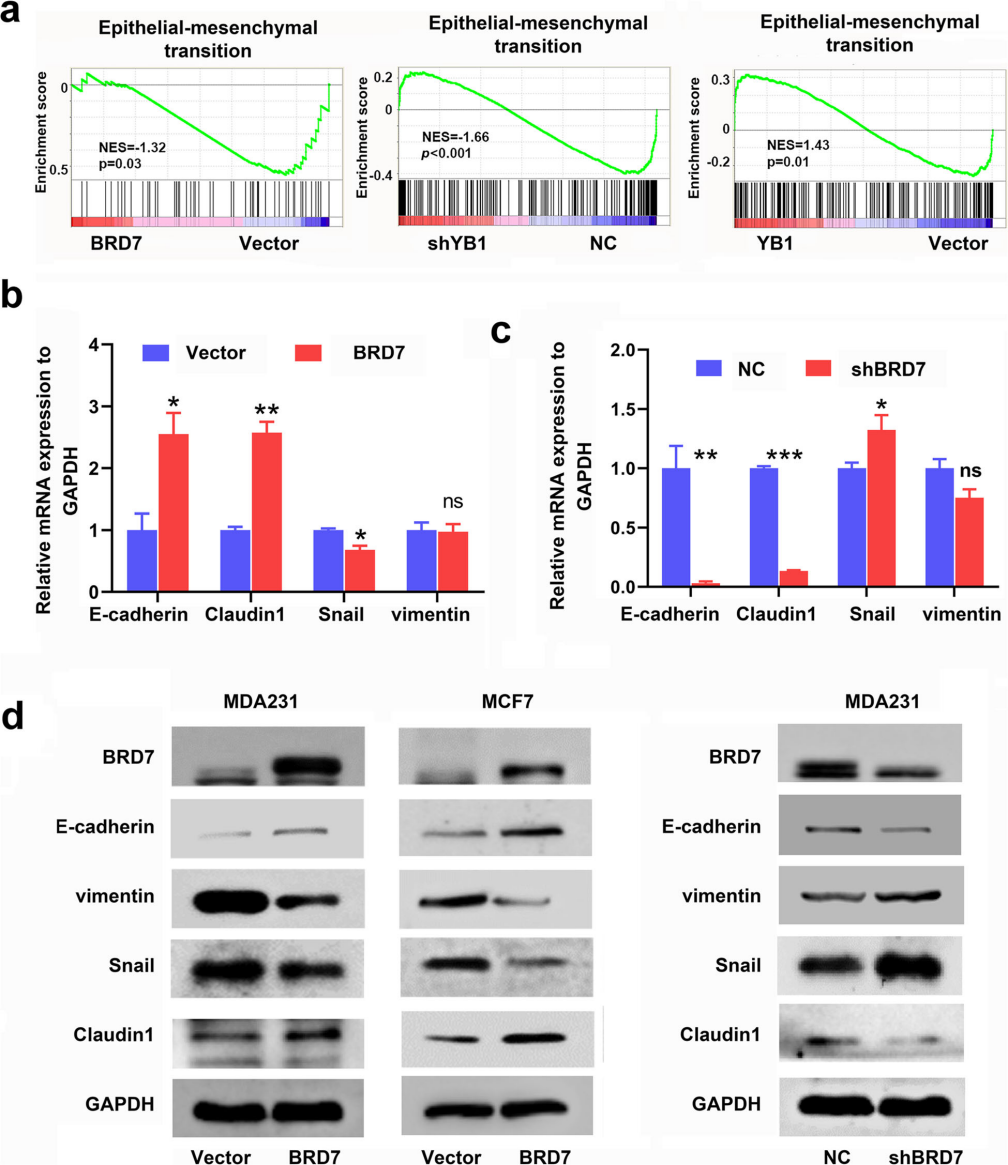
BRD7 inhibits the process of EMT. **a** GSEA analysis of microarray data from BRD7 overexpressed (left), YB1 knockdown (middle) or YB1 overexpressed cells (right) and control. **b** qPCR analysis of E-cadherin, Claudin1, vimentin and Snail in MDA231 cells with BRD7 overexpression. Data represent means ± SEMs. ns, *p* > 0.05; *, *p* < 0.05; **, *p* < 0.01; ***, *p* < 0.001. **c** qPCR analysis of E-cadherin, Claudin1, vimentin and Snail in MDA231 cells with BRD7 inhibition. Data represent means ± SEMs. ns, *p* > 0.05; *, *p* < 0.05; **, *p* < 0.01; ***, *p* < 0.001. **d** Immunoblots of BRD7, E-cadherin, Claudin1, vimentin and Snail in MDA231 and MCF7 cells with BRD7 overexpression or in MDA231 cells with BRD7 knock-down

EMT is an important process of invasion and metastasis. Cells with a high invasion ability have a mesenchymal cell morphology, while cells with a low invasion ability have an epithelioid morphology. Surprisingly, we discovered that cells are prone to acquire an epithelial-like morphological phenotype after BRD7 overexpression (Additional file 1: Figure S4a). To identify the molecular events induced by BRD7 and YB1 in breast cancer cell invasion, we revisited our RNA-seq data (PRJNA562788) and the public database (GSE60964 and GSE6562) by GSEA. The results showed that the molecular expression is aggregated in the process of EMT after overexpression of BRD7 or YB1 or depletion of YB1 (Fig. 4a). To test the effect of BRD7 on EMT, we measured the mRNA and protein levels of epithelial molecular markers such as E-cadherin and Claudin1 and mesenchymal molecular markers such as Snail and vimentin. The qRT-PCR and western blotting results demonstrated that BRD7 overexpression reduced the abundance of Snail and promoted the expression of E-cadherin and Claudin1. BRD7 shRNA had an opposite effect in MDA231 cells. The protein expression of vimentin was decreased in BRD7-overexpressing cells and increased in BRD7-depleted MDA231 cells, whereas vimentin mRNA expression was not markedly affected (Fig. 4b-d). We then examined vimentin expression utilizing immunofluorescence and found that the fluorescence intensity of vimentin was weaker in BRD7-overexpressing cells than in control cells (Additional file 1: Figure S4B). In agreement with a previous study [20], YB1 overexpression caused an increase in Snail expression and a decrease in E-cadherin expression (Additional file 1: Figure S5a). Conversely, YB1 knockdown obviously downregulated Snail and vimentin expression and upregulated E-cadherin expression (Additional file 1: Figure S5b). Collectively, the changes in cell morphology and the expression of the relevant molecular markers indicate that cells with a high level of BRD7 undergo mesenchymal-epithelial transition (MET) and display a predominantly epithelial phenotype.

### Restoring the expression of YB1 abrogated the inhibitory effect of BRD7 on cell growth and migration

**Fig. 5:**
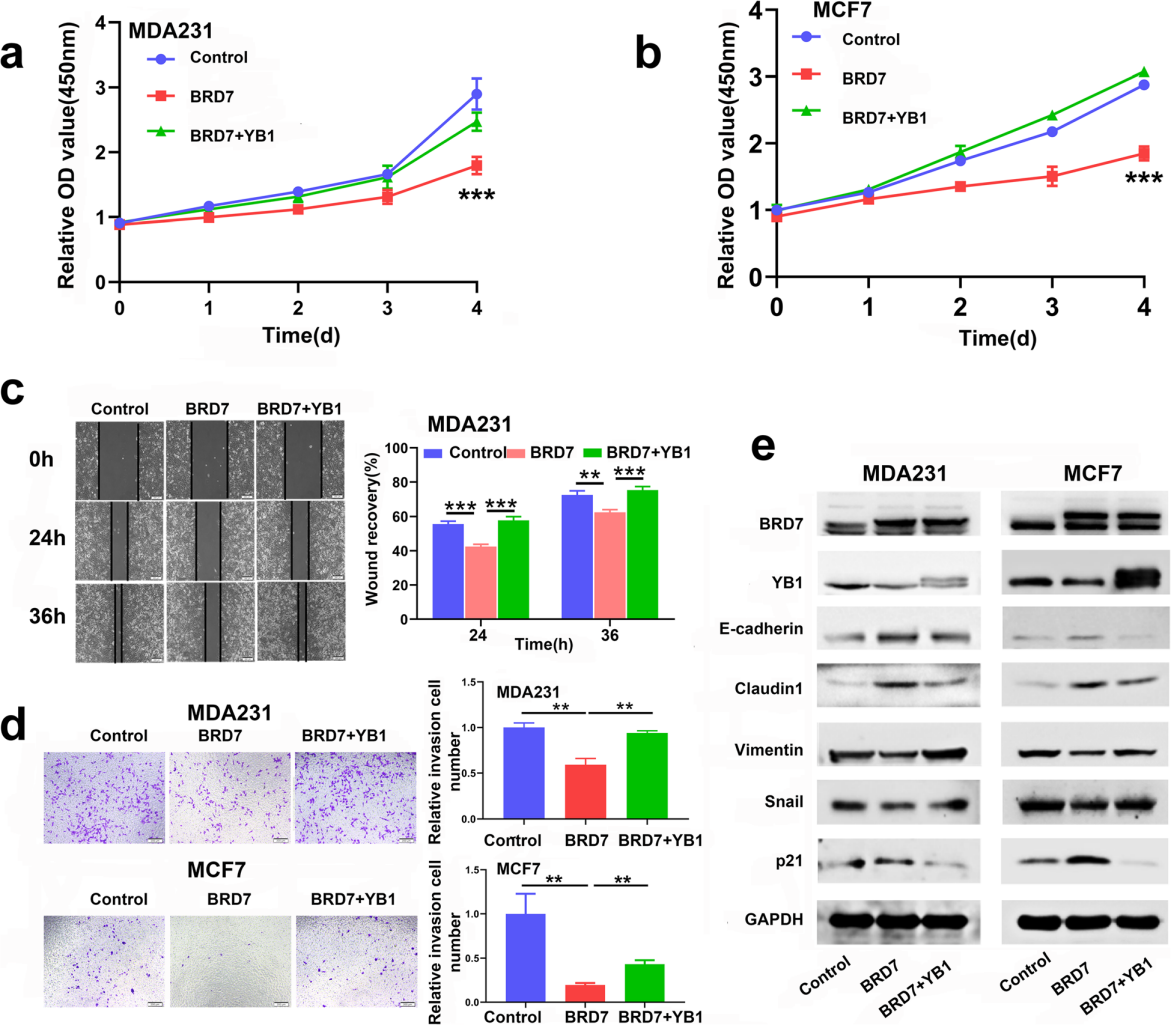
YB1 antagonizes the inhibitory effect of BRD7 on cell proliferation, migration and invasion. **a** and **b** CCK8 analysis of cell proliferation in MDA231 and MCF7 cells stably with BRD7 overexpression, BRD7 and YB1 simultaneous overexpression or control group. Data represent means ± SDs. *, *p* < 0.01. **c** Scratch wound healing analysis of cell migration in MDA231 cells with BRD7 overexpression, BRD7 and YB1 simultaneous overexpression or control. Quantification of wound recovery rate of the three groups (right). Data represent means ± SEMs. **, *p* < 0.01; ***, *p* < 0.001. **d** Matrigel invasion analysis of cell invasive capabilities in MDA231 and MCF7 cells with BRD7 overexpression, BRD7 and YB1 simultaneous overexpression or control. Data represent means ± SDs. **, *p* < 0.01. **e** Western blotting analysis of the expression of BRD7, YB1, E-cadherin, Claudin1, vimentin, Snail and p21 in BRD7 overexpression and YB1 restoration cells

Over the past decades, substantial efforts have been directed at the key role played by YB1 in the process of tumor metastasis [20], and our above results showed that BRD7 not only produces a suppressive effect on the migration and invasion of breast cancer cells but also promotes the degradation of the YB1 protein. Therefore, we sought to determine whether YB1 plays an essential role in the BRD7-mediated tumor suppressor function. Therefore, we set up a series of rescue experiments to test this hypothesis. CCK8 assay showed that BRD7 overexpression significantly inhibited the growth of breast cancer cells compared with control cells but restoration of YB1 expression recovers the inhibitory effect of BRD7 on cell proliferation in MDA231 and MCF7 cells (Fig. 5a and b). Next, scratch wound healing assays and Matrigel invasion assays revealed that ectopic expression of BRD7 suppressed cell migration and cell invasion, while YB1 restoration rescued the capabilities of cell migration and cell invasion (Fig. 5c and d). A set of markers can highlight the status of cells during the process of EMT, in which the expression of these molecules is decreased or increased. It is known that YB1 can trigger EMT in a cap-independent translation manner [20]. To further investigate the effect of YB1 restoration on BRD7-mediated EMT, we monitored the expression of the epithelial molecular markers E-cadherin and Claudin1 as well as the mesenchymal molecular markers vimentin and Snail after YB1 restoration. As expected, upregulation of E-cadherin, Claudin1 and p21 and downregulation of vimentin and Snail were found in BRD7-overexpressing cells, while the expression levels of these EMT-related markers were significantly reversed, at least partly, after YB1 restoration (Fig. 5e). These data thus suggest that YB1 might play a leading role in BRD7-mediated growth, migration, invasion and EMT of breast cancer cells.

### YB1 impairs the antagonistic effect of BRD7 on tumorigenesis in vivo

**Fig. 6:**
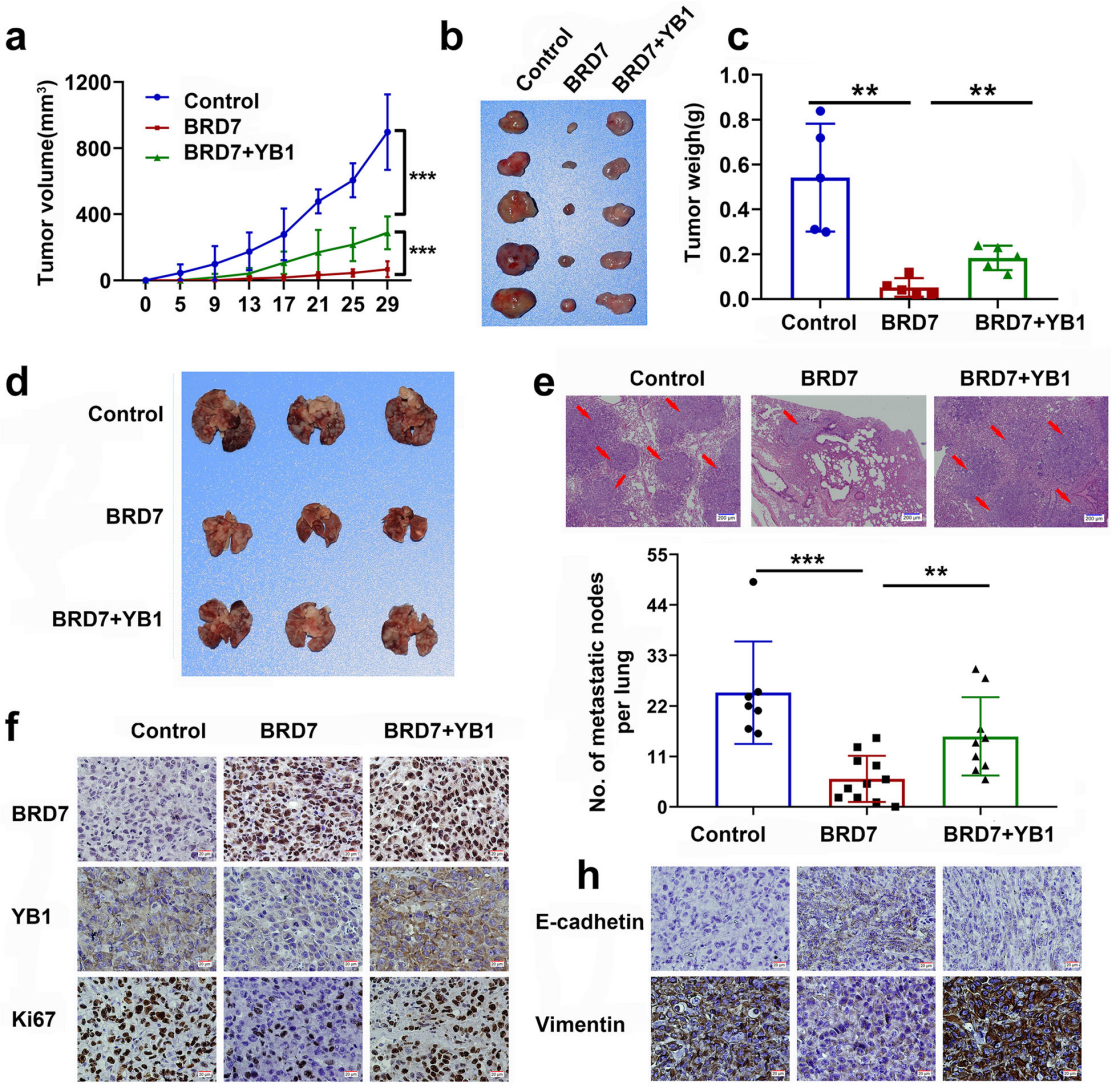
BRD7 suppresses tumor growth and reduces lung metastasis through YB1 in vivo. **a**, **b** and **c** Tumor volume, image and tumor weight of nude mice with MDA231 cells in xenograft model, *n* = 5 mice per group. Data represent means ± SDs. **, *p* < 0.01. **d** Representative image of macroscopic mouse lung tissue in the metastatic tumor model, *n* = 11 mice per group. **e** Representative image of lung metastasis samples by H&E staining is shown in control, BRD7 overexpression and YB1 restoration group. Red arrows indicate metastatic tumors, scale bar, 200 μm. The number of metastatic lung nodules of every mouse per group were counted in microscopy. Data represent means ± SDs. **, *p* < 0.01; ***, *p* < 0.001. **f** Primary tumor samples for IHC analysis of the expression of BRD7, YB1, Ki67 in control, BRD7 overexpression and YB1 restoration group, scale bar, 20 μm. **h** Primary tumor samples for IHC analysis of the expression of EMT markers E-cadherin and vimentin, scale bar, 20 μm

Our above work confirmed that YB1 can antagonize the inhibitory effect of BRD7 on EMT. To further explore the molecular mechanism, we established xenograft and metastasis models using MDA231 cells in three groups of mice: the control, BRD7 and YB1 recovery groups. For the xenograft model, 3 × 10^6^ MDA231 cells were inoculated subcutaneously into the left shoulders of nude mice. Tumors began to grow on the 5th day. Tumors were measured once every 4 days, and all mice were sacrificed on day 29. The results showed that BRD7 overexpression significantly inhibited tumor growth and that the tumor weight was lower in the BRD7 overexpression group than in the control group, but the tumor weight was recovered after YB1 restoration (Fig. 6a, b and c and Additional file 1: Figure S6a). For the metastatic tumor model, 2 × 10^6^ MDA231 cells were injected intravenously to generate lung metastases. All animals were sacrificed on day 31, and lung tissue was then removed, photographed, embedded in paraffin and stained with HE. Interestingly, we found the metastatic lung nodules of nude mice in BRD7 overexpression group are significantly less than those in control, which partly increased in YB1 restoration group (Fig. 6d and Additional file 1: Figure S6b), and the result of HE staining of lung metastasis samples was consistent with this result (Fig. 6e). These results indicate that BRD7 significantly dampens lung metastasis of breast cancer in vivo, in accordance with the in vitro results. Notably, IHC of primary tumor samples was used to detect the alterations of BRD7, YB1, Ki67, E-cadherin and vimentin. The results showed that BRD7 was successfully overexpressed, the expression of YB1 was decreased in the BRD7-overexpression group, and YB1 expression was successfully restored. Fewer Ki67-positive cells were observed in the BRD7 overexpression group than in the control group; and the expression of vimentin decreased and the expression of E-cadherin increased, while these changes were partially reversed as a consequence of YB1 restoration (Fig. 6f and h). The results in vivo and in vitro indicate that BRD7 inhibits tumor growth and lung metastasis in breast cancer through the regulation of YB1.

### BRD7 and YB1 are negatively correlated in breast cancer prognosis

**Fig. 7:**
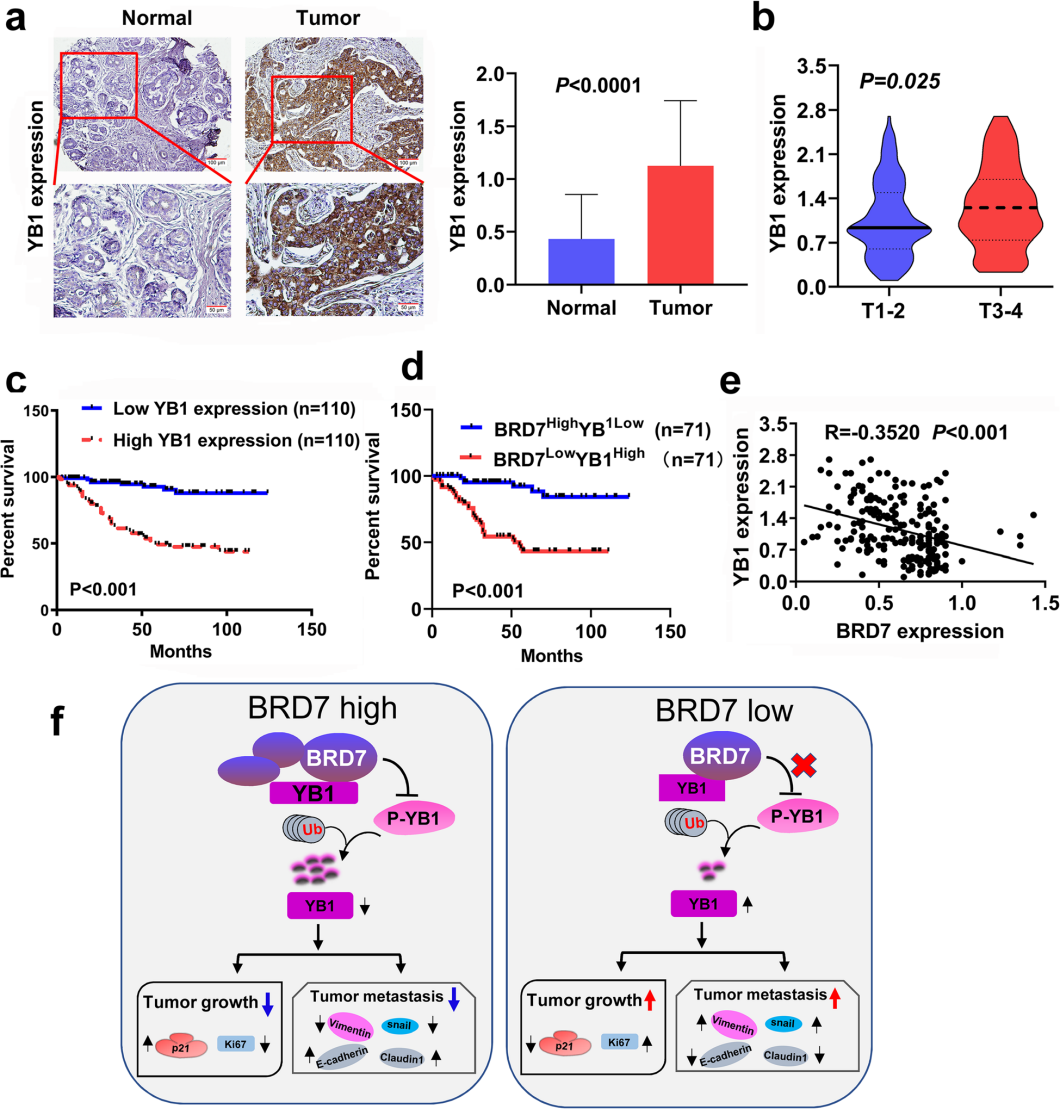
BRD7 is negatively correlated with YB1 in breast cancer. **a** YB1 expression was determined in normal (*n* = 43) and tumor samples (*n* = 220) by IHC. **b** YB1 expression in different T stages of breast cancer. **c** and **d** Kaplan-Meier curves showed the overall survival of breast cancer patients. High or low expression of YB1, and low BRD7 plus high YB1 level and high BRD7 plus low YB1 level. **e** The correlation between BRD7 and YB1 was performed based on chi-square test. **f** Schematic representation of molecular mechanism of BRD7 in suppressing tumor growth and metastasis

The results of our in vitro and in vivo experiments confirmed a negative correlation between BRD7 and YB1. Considering our previous finding that BRD7 is lowly expressed and positively correlated with prognosis in breast cancer samples [8], which is consistent with the results of Nigro et al. [7], To gain a more general insight into the association between BRD7 and YB1 in clinical specimens, we used IHC to examine the molecular expression level of YB1 in a total of 220 human breast cancer samples and 43 normal breast samples. The results showed that YB1 was highly expressed in breast cancer, that its expression in clinical stages 3 and 4 was significantly higher than that in stages 1 and 2, and that high levels of YB1 are correlated with poor clinical outcomes of breast cancer patients (Fig. 7a, b and c). Moreover, an unfavorable overall survival was observed in breast cancer patients with a combination of low BRD7 expression and high YB1 expression (Fig. 7d). There was a negative correlation between BRD7 and YB1 with an *R* value of − 0.3520 (Fig. 7e). Statistical analysis of clinical patients showed that high YB1 expression and low BRD7 expression combined with high YB1 expression were both correlated with tumor size, distant metastasis, TNM stage, ER and PR and that the difference was more statistically significant in samples with low BRD7 expression combined with high YB1 expression (Table 2). These results suggest that BRD7 is negatively correlated with YB1 and low BRD7 combined with high YB1 levels might be a marker of poor prognosis in breast cancer patients.

**Table 2:** The association between BRD7, YB1 expression and clinicopathologic features of breast cancer

Variables	YB1 expression			BRD7/YB1 expression		
Features	YB1^H^	YB1^L^	*P*	BRD7^L^YB1^H^	BRD7^H^YB1^L^	*P*
Age (Y)						
<46	50 (45.5)	56 (50.9)	0.418	35 (49.3)	32(45.1)	0.737
≥46	60 (54.5)	54 (49.1)		36 (50.7)	39(54.9)	
Tumor size (%)						
T1~2	50 (45.5)	71 (64.5)	0.008**	31 (43.7)	46 (64.8)	0.020*
T3~4	60 (54.5)	39 (35.5)		40 (56.3)	25 (35.2)	
Nodal metastasis (%)						
Absent	23 (20.9)	25 (22.7)	0.567	15 (21.1)	19 (26.8)	0.432
Present	87 (79.1)	85 (77.3)		56 (78.9)	52 (73.2)	
Distant metastasis (%)						
Absent	94 (85.5)	106 (96.4)	0.005**	60 (84.5)	69 (97.2)	0.009**
Present	16 (14.5)	4 (3.6)		11 (15.5)	2 (2.8)	
TNM stage (%)						
I~II	65 (59.1)	84 (76.4)	0.018*	41 (57.7)	55 (77.5)	0.038*
III~ IV	45 (40.9)	26 (23.6)		30 (42.3)	16 (22.5)	
ER expression (%)						
Negative	57 (51.8)	35 (32.4)	0.004**	46 (64.8)	21 (30.4)	0.000***
Positive	53 (48.2)	73 (67.6)		25 (35.2)	48 (69.6)	
PR expression (%)						
Negative	58 (52.7)	37 (34.3)	0.006**	45 (63.4)	23 (33.3)	0.000***
Positive	52 (47.3)	71 (65.7)		26 (36.6)	46 (66.7)	
HER2 expression (%)						
Negative	39 (35.5)	40 (37.0)	0.808	25 (35.2)	29 (42.0)	0.407
Positive	71 (64.5)	68 (63.0)		46 (64.8)	40 (58.0)	

In 220 patients, ER, PR and HER2 expression was not detected in two of the patients with low YB1 expression, and the median of YB1 or BRD7 expression was used as the cutoff value

*Y* Year, *TNM* Tumor-node-metastases, *H* High expression, *L* Low expression, *P P* values of two-sided χ2 test, *%* The ratio of the number of samples to the total number of samples per column, * *p* < 0.05, ** *p *< 0.01, *** *p *< 0.001

## Discussion

As a member of the bromodomain-containing protein family, BRD7 contributes to the inhibition of cell proliferation and cell cycle progression and to the induction of apoptosis in several types of cancers, including NPC and breast cancer [6–8, 12, 22]. We previously confirmed that BRD7 plays an inhibitory effect on cell cycle progression by inhibiting the nuclear translocation of β-catenin and the activation of the ERK1/2 pathway in NPC, thus blocking tumor growth [13]. Recent one study showed that BRD7 inhibits tumor growth, invasion and metastasis and induces apoptosis in epithelial ovarian carcinoma by negatively regulating the β-catenin pathway [16]. BRD7, a coactivator of p53, directly binds with p53, is recruited to the promoter regions of p53 target genes, and is involved in the regulation of downstream target genes of p53 such as p21 and HDM2 [14]. In agreement with these results, we showed that BRD7 inhibits cell proliferation as well as cell migration, invasion and metastasis through in vitro and in vivo experiments. To our knowledge, this is the first report on the association of BRD7 with tumor invasion and metastasis in breast cancer. These results support the hypothesis that BRD7 inhibits tumorigenesis and metastasis and thus plays a critical antioncogenic role in breast cancer.

An increasing number of studies have confirmed that EMT is pathologically reactivated and plays a pivotal role in the tumorigenic process [2]. E-cadherin deficiency is an important molecular marker of EMT in tumor cells. Snail and Slug, markers of mesenchymal phenotypes, negatively regulate the expression of E-cadherin at the transcriptional level [32]. And Snail can also inhibit the expression of other epithelial genes such as Claudin1 and Muc1 and promote the expression of other mesenchymal genes such as fibronectin, MMP9 and vimentin, which activates EMT and is related to tumor metastasis, recurrence and poor prognosis in breast cancer [33–35]. In view of the morphological and molecular changes that occur during the EMT process, we examined these changes after BRD7 overexpression. Elevated levels of BRD7 maintained the morphology of epithelial cells and blocked the morphological transformation to mesenchymal cells. In addition, BRD7 increased the expression of epithelial molecules such as E-cadherin and Claudin1 and decreased the expression of mesenchymal molecules such as Snail and vimentin in breast cancer cells. Importantly, ectopic expression of BRD7 inhibited cell proliferation, migration, invasion and metastasis. Overall, our data suggest that BRD7 might inhibit cell migration, invasion and metastasis through negatively regulating the EMT process in breast cancer.

To further explore the specific molecular mechanism by which BRD7 is involved in breast cancer invasion and metastasis, we screened the proteins interacting with BRD7. As a result, YB1 was identified as a new interacting protein of BRD7. Surprisingly, ectopic expression of BRD7 decreased the expression of YB1 at the protein level. Earlier studies showed that YB1 can regulate tumor growth and metastasis via transcriptional regulation of EGFR, HER2, MDR1, TP53 and AP1 through its Y-box or other YB1 response element [36]. Apart from its transcriptional regulation function, YB1 translationally activates a set of mRNAs whose protein products are involved in the process of embryonic development and tumor progression, such as Snail, twist, HIF1a and MYC [37–39]. For instance, YB1 activates Snail by directly binding its mRNA in a cap-independent translational manner promoting EMT [40]. Here, our findings suggest that YB1 increases the expression of Snail and vimentin and decreases the expression of E-cadherin. Furthermore, restoration of YB1 in BRD7-overexpressing cells partially recover the inhibitory effect of BRD7 on cell migration and invasion, as well as the expression of E-cadherin, Claudin1, Snail and vimentin. Therefore, an intriguing possibility is that BRD7 may prevent YB1-mediated translational regulation of Snail in a cap-independent translational manner, thereby promoting the acquisition of epithelial-like properties and restricting metastatic progression. Moreover, a previous study showed that BRD7 cooperates with p53 to suppress the expression of p21 and HDM2 at the transcriptional level [14]. Recent evidence has suggested that YB1, an interacting protein of the lncRNA MIR22HG, strongly increases MET expression and decreases p21 expression to regulate cell proliferation, apoptosis and senescence [41]. We observed that p21 protein levels were increased in the BRD7 overexpression group but dramatically decreased after YB1 restoration in our experimental system, suggesting that BRD7 exerts antiproliferative effects through YB1-mediated inhibition of p21. Therefore, the present study provides corroborating evidence that BRD7 inhibits cell proliferation, EMT and metastasis through YB1-mediated induction of tumor growth and metastasis.

YB1 plays a key role in the antitumor function of BRD7, and our further studies showed that BRD7 decreases YB1 phosphorylation at Ser102. It is noteworthy that a majority of kinases in the AKT/mTOR and MEK/ERK signaling pathways can activate YB1 phosphorylation at Ser102, thus promoting the activation of drug-resistant genes and genes associated with malignant phenotypes [28, 42]. The phosphorylation of YB1 at Ser102 is associated with migratory and invasive activity in breast cancer and melanoma [21, 40]. Our previous results confirmed that BRD7 negatively regulates the AKT signaling pathway to inhibit cell proliferation and tumor formation [12]. In this study, we demonstrated that BRD7 interacts with YB1 and negatively regulates the YB1 phosphorylation level. As a multifunctional protein, YB1 is cleaved into a truncated protein in the middle of the YB1 CTD through the proteasome pathway in response to regulatory genes or multiple drugs, such as cisplatin and Taxol [43]. The E3 ubiquitin ligases FBX33 [43] and RBBP6 [44] and long non-coding RNA MIR22HG [41] could interact with YB1 to induce its ubiquitination and proteasomal degradation. Our results showed that BRD7 interacts with YB1 and downregulates the protein expression of YB1 by inducing its ubiquitin-mediated degradation. More strikingly, an increasing number of works have identified that substrate phosphorylation induces conformational changes that contribute to ubiquitin-mediated proteasomal degradation. For example, phosphorylation of newly synthesized c-Myc protein at Ser62 enhances its stability. Thr58 phosphorylation of c-Myc promotes Ser62 dephosphorylation and is required for c-myc degradation [45]. Phosphorylation of Bim-EL at Ser 69 is required for its proteasomal degradation [46]. The phosphorylation status of YB1 itself is very important for its function. Abolition of YB1 phosphorylation at Ser102 or mutation of S102 to Ala blocks the nuclear translocation, DNA-binding ability and translation of the YB1 protein [47]. Notably, an important finding of this study is that BRD7 appreciably inhibits YB1 phosphorylation at Ser102, which is vital for its proteasomal degradation. As emphasized, our results show that BRD7 obviously decreases the expression and phosphorylation levels of YB1, thereby inducing the proteasomal degradation of YB1.

Many reports have shown that YB1 is widely overexpressed in tumors and is an independent prognostic factor. And previous evidences confirm that the 5-year survival rate in breast cancer patients with low expression of YB1 was about 90% [19, 48, 49]. Consistent with these findings, we found that high expression of YB1 is observed in breast cancer and is correlated with tumor growth and distant metastasis. A negative correlation exists between BRD7 and YB1, and the combination of low BRD7 expression and high YB1 expression is significantly associated with poor prognosis and metastasis. Therefore, it is worthwhile to further explore the clinical application of BRD7 and YB1 in breast cancer.

## Conclusions

We conclude that BRD7 plays an essential role in tumorigenesis and metastasis by negatively regulating YB1-mediated EMT. BRD7 interacts with YB1 and inhibits its phosphorylation at Ser102, thus leading to ubiquitination-mediated degradation of YB1. Low BRD7 expression combined with high YB1 expression is significantly correlated with poor prognosis, distant metastasis and advanced TNM stage. The developmental mechanism of BRD7-mediated malignant features could be helpful for designing personalized treatments for breast cancer.

## Supplementary information

**Additional file 1: Figure S1.** Knockdown of BRD7 induces cell migration and cell invasion in breast cancer cells. **Figure S2.** BRD7 colocalized with YB1 in MCF7 and HEK293T cells. **Figure S3.** YB1 has no effect on BRD7 mRNA and protein level. **Figure S4.** BRD7 inhibits EMT processs and vimentin expression. **Figure S5.** YB1 induces the expression of Snail and vimentin and reduces E-cadherin expression in breast cancer cells. **Figure S6.** BRD7 inhibits tumor growth and reduces lung metastasis.

## Data Availability

All data generated or analyzed during this study are included in this published article and its supplementary information files.

## Abbreviations

ACN: Acetonitrile
BCA: Bicinchoninic acid
BRCA: Breast cancer
DMEM: Dulbecco’s Modified Eagle’s Medium
EMT: Epithelial-mesenchymal transition
FBS: Fetal bovine serum
HE: Hematoxylin-eosin staining
IHC: Immunohistochemical staining
MET: Mesenchymal-epithelial transition
RPMI: Roswell Park Memorial Institute
YB1: Y-box binding protein-1
