# Supplementary material for: BRD7 suppresses invasion and metastasis in breast cancer by negatively regulating YB1-induced epithelial-mesenchymal transition
Source: J Exp Clin Cancer Res. 2020 Feb 7;39:30. doi: 10.1186/s13046-019-1493-4 (PMC7006413; doi:10.1186/s13046-019-1493-4)
Supplement: Supplementary file 1 — Additional file 1: Figure S1. Knockdown of BRD7 induces cell migration and cell invasion in breast cancer cells. Figure S2. BRD7 colocalized with YB1 in MCF7 and HEK293T cells. Figure S3. YB1 has no effect on BRD7 mRNA and protein level. Figure S4. BRD7 inhibits EMT processs and vimentin expression. Figure S5. YB1 induces the expression of Snail and vimentin and reduces E-cadherin expression in breast cancer cells. Figure S6. BRD7 inhibits tumor growth and reduces lung metastasis. [file 13046_2019_1493_MOESM1_ESM.docx]

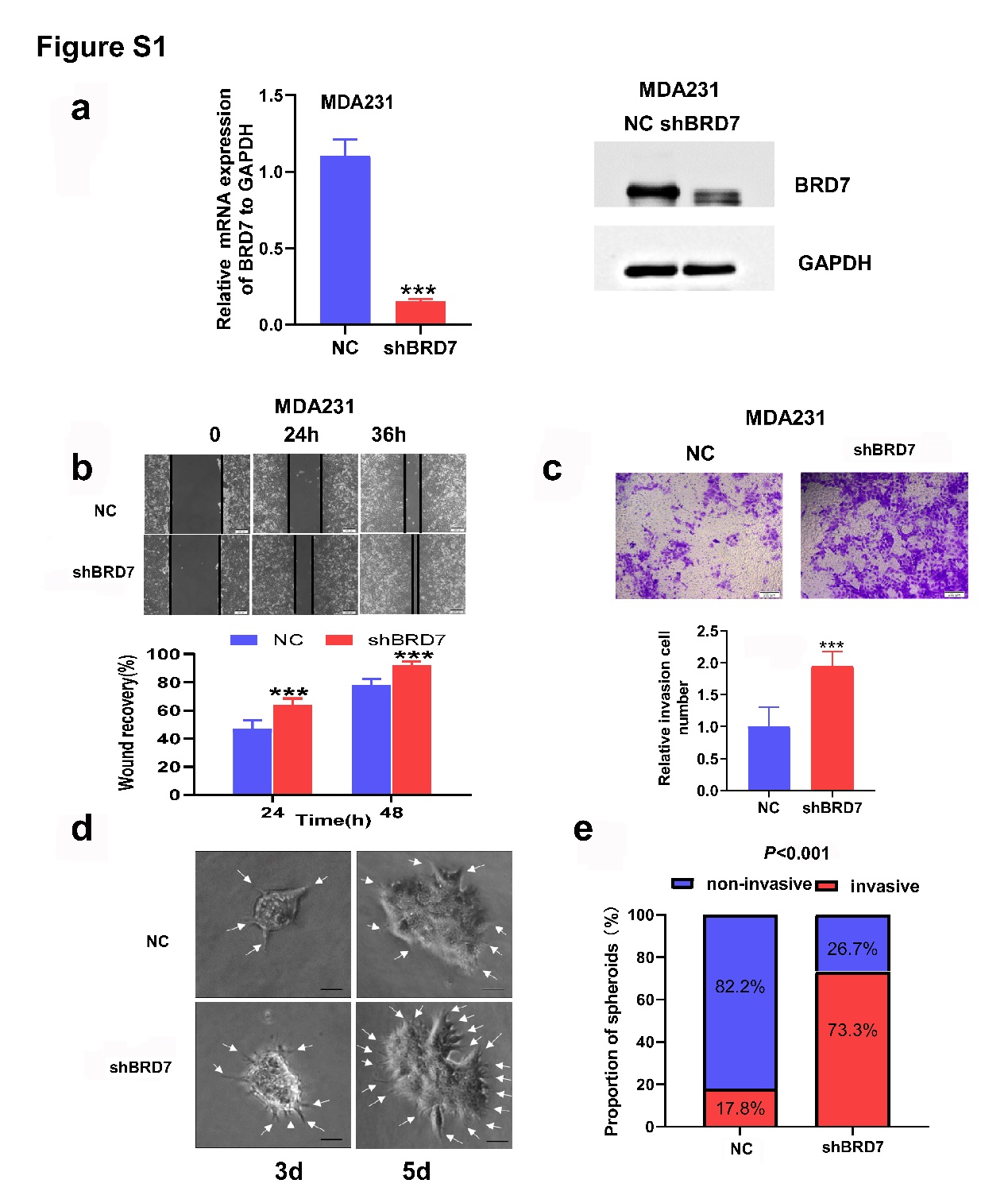


**Figure S1** Knockdown of BRD7 induces cell migration and cell invasion in breast cancer cells. (A) qPCR and Western blotting analysis of BRD7 expression in MDA231 cells stably transfected with BRD7 shRNA or NC. Data represent means ± SDs. ***, p < 0.001. (B and C) scratch wound healing and matrigel invasion respectively analysis cell migration and invasion in BRD7 shRNA or NC group. Data represent means ± SDs. ***, p < 0.001. (D) Three-dimensional invasion analysis of cell invasive capabilities after BRD7 silenced, scale bar, 50 μm. (E) Quantification of invasive and non-invasive clonal spheroids in BRD7 shRNA or NC group. Data represent means ± SDs.

**
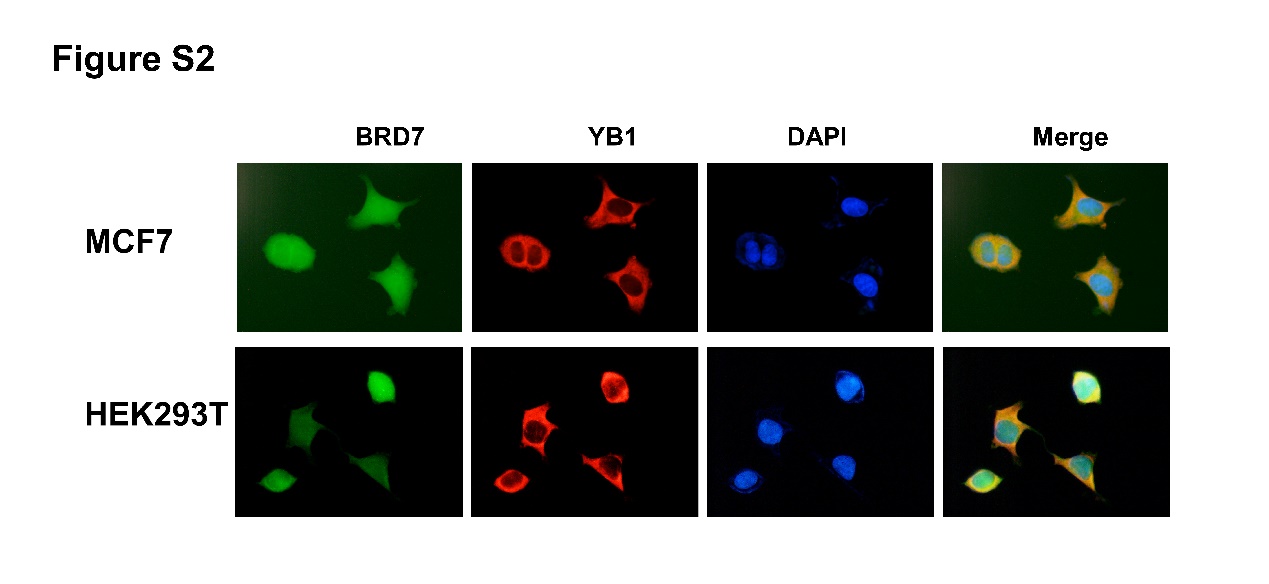
**

**Figure S2** BRD7 colocalized with YB1 in MCF7 and HEK293T cells.

Immunofluorescence assay was performed in flag-BRD7 overexpressed MCF7 and HEK293T cells by using anti-flag and anti-YB1 antibodies.


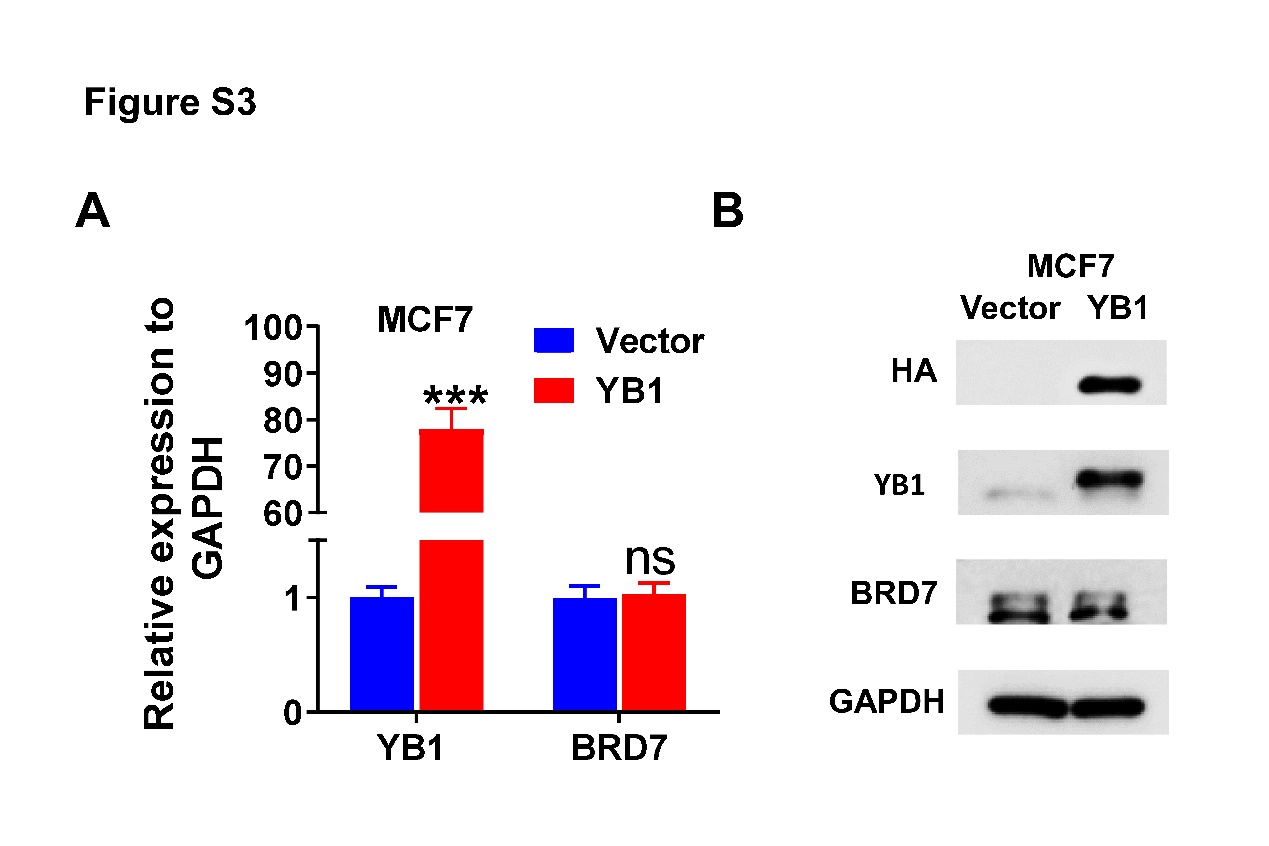


**Figure S3** YB1 has no effect on BRD7 mRNA and protein level. (A) qRT-PCR analysis of YB1 and BRD7 in YB1 overexpressed MCF7 cells. Data represent means ± SDs. ns, p >0.05; ***, p < 0.001. (B) Western blotting analysis of YB1 and BRD7 in YB1 overexpressed MCF7 cells.


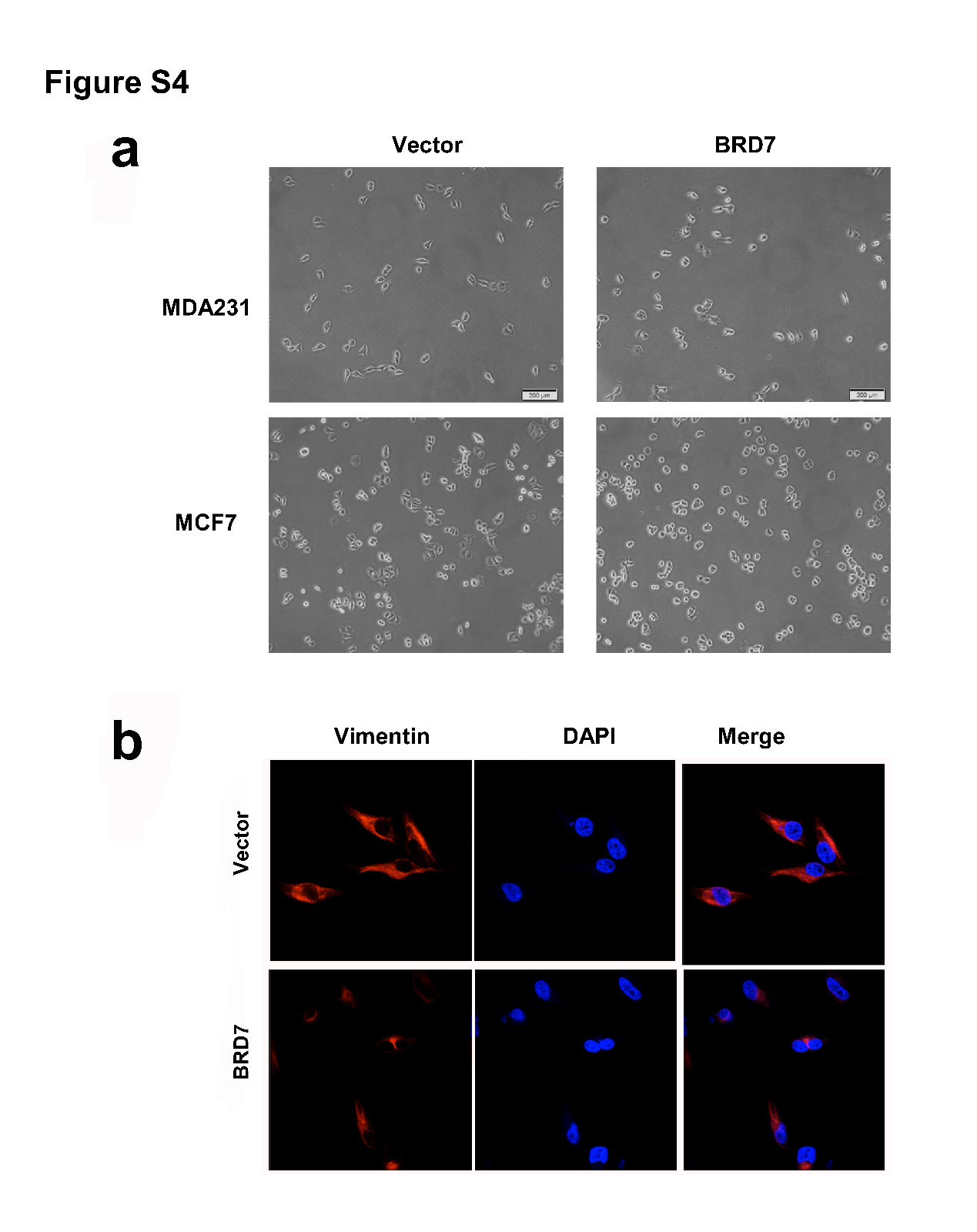


**Figure S4** BRD7 inhibits EMT process and vimentin expression. (A) Cell morphology of BRD7 overexpressed MCF7 and MDA231 cells. (B) Immunofluorescence analysis of vimentin in BRD7 overexpressed MDA231 cells.


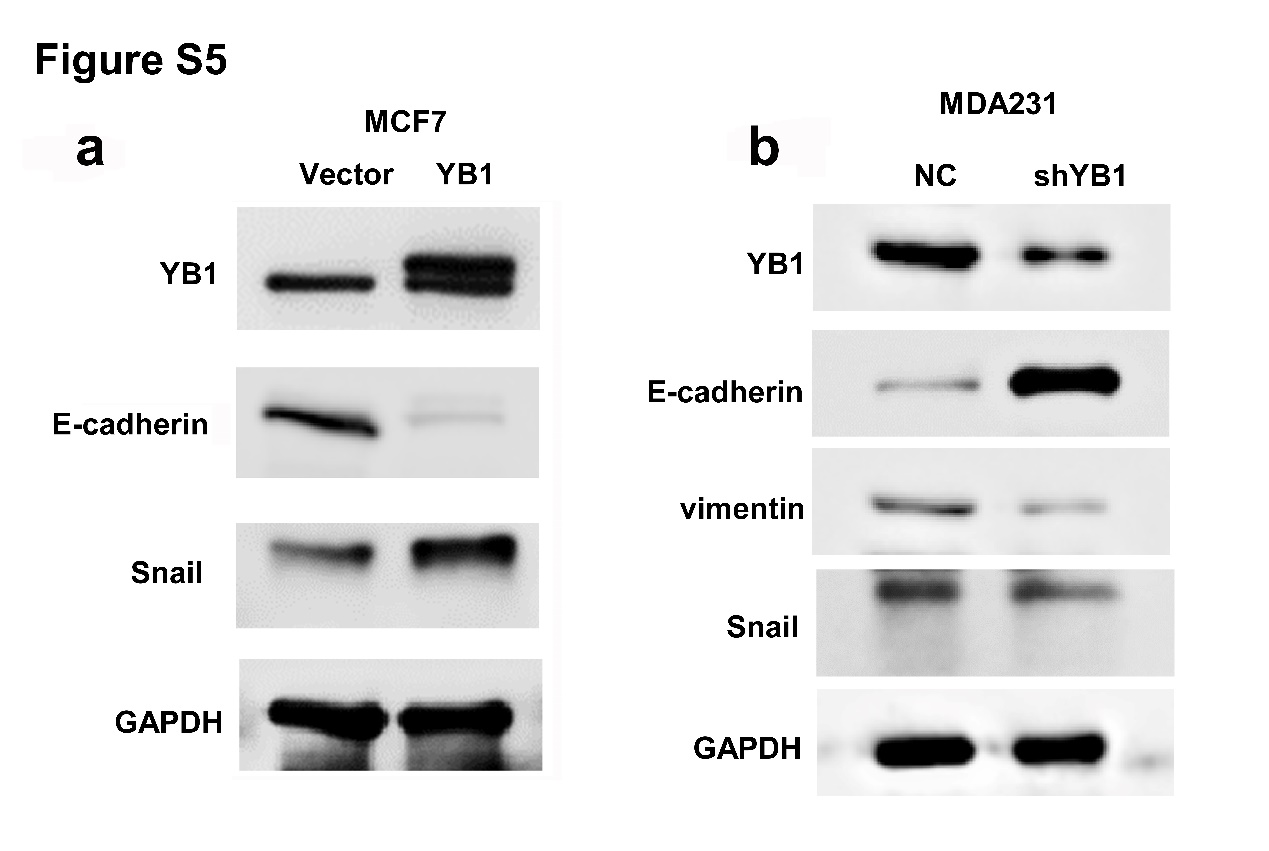


**Figure S5** YB1 induces the expression of Snail and vimentin and reduces E-cadherin expression in breast cancer cells. (A) Immunoblots of YB1, E-cadherin, Snail in MCF7 cells with YB1 overexpression. (B) Immunoblots of YB1, E-cadherin, vimentin and Snail in MDA231 cells with YB1 knock-down.


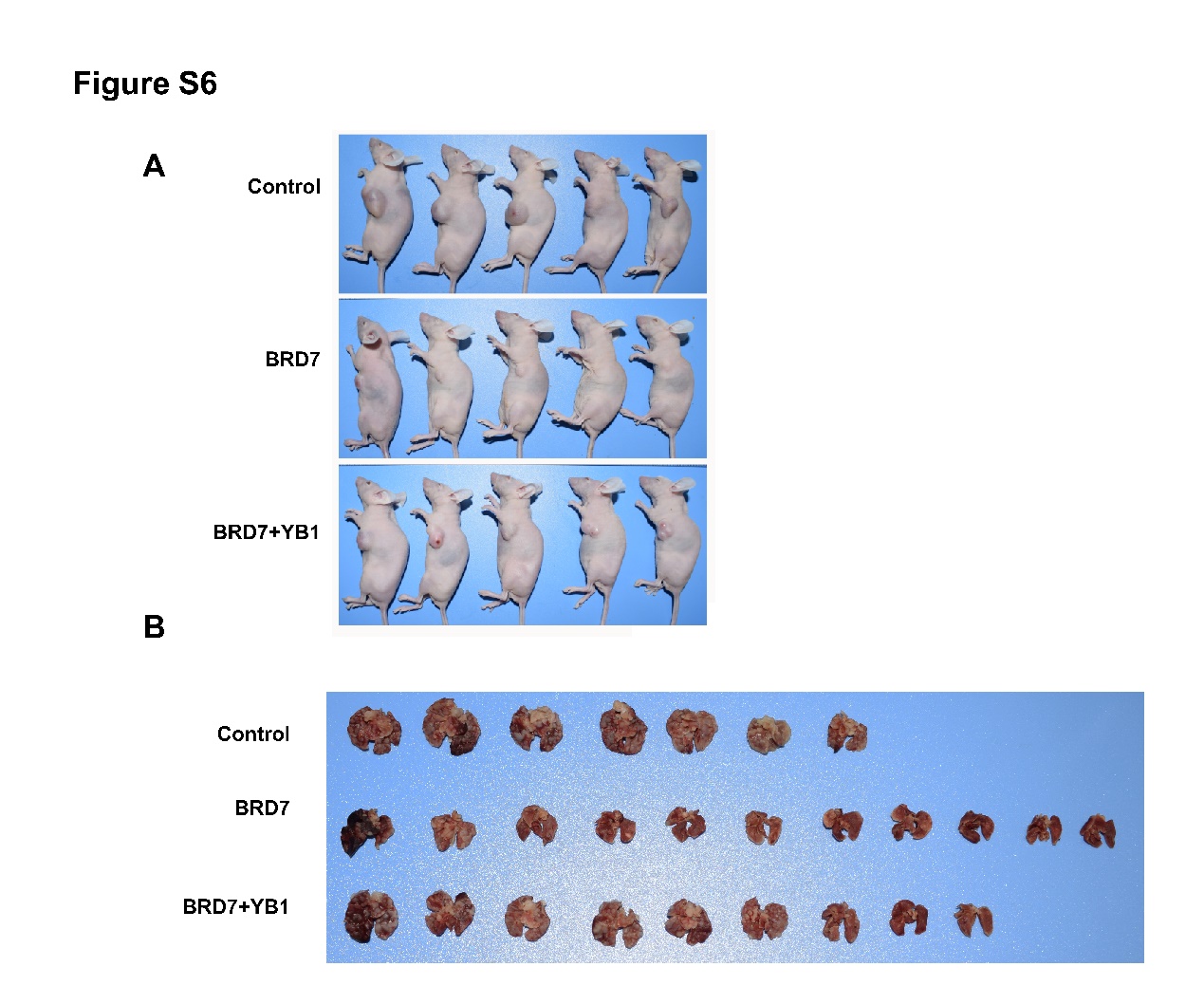


**Figure S6** BRD7 inhibits tumor growth and reduces lung metastasis.

(A) Macroscopic mouse of xenograft model, n=5 per group. (B) Macroscopic mouse lung of metastatic tumor model, n=11 per group. At the middle stage of the experiment, 4 mice died in the control group and 2 mice died in the BRD7 plus YB1 group.
